# Recent Advances in the Application of 2‐Aminobenzothiazole to the Multicomponent Synthesis of Heterocycles

**DOI:** 10.1002/open.202400185

**Published:** 2024-09-09

**Authors:** Ramin Javahershenas, Jianlin Han, Mosstafa Kazemi, Peter J. Jervis

**Affiliations:** ^1^ Department of Organic Chemistry Faculty of Chemistry Urmia University Urmia Iran; ^2^ Jiangsu Co-Innovation Center of Efficient Processing and Utilization of Forest Resources College of Chemical Engineering Nanjing Forestry University Nanjing 210037 China; ^3^ Young Researchers and Elite Club Tehran Branch Islamic Azad University Tehran Iran; ^4^ Center of Chemistry University of Minho Campus de Gualtar 4710-057 Braga Portugal

**Keywords:** 2-aminobenzothiazole, heterocycles, multicomponent reactions, synthesis

## Abstract

Heterocycles are a vital class of compounds in numerous fields, including drug discovery, agriculture, and materials science. Efficient methods for the synthesis of heterocycles remain critical for meeting the demands of these industries. Recent advances in multicomponent reactions (MCRs) utilizing 2‐aminobenzothiazole (ABT) have shown promising results for the formation of heterocycles. The versatility of 2‐aminobenzothiazole in this context has enabled the rapid and efficient construction of diverse heterocyclic structures. Various synthetic methodologies and reactions involving 2‐aminobenzothiazole are discussed, highlighting its importance as a valuable building block in the synthesis of complex heterocycles. The potential applications of these heterocycles in drug discovery and material science are also explored. Overall, this review provides a comprehensive overview of the current state of research in the field and offers insights into the future directions of this promising area of study. We highlight the potential of ABT as a versatile and sustainable starting material in heterocyclic synthesis via MCRs, with significant implications for the chemical industry.

## Graphical abstract

This review highlights recent advances in multicomponent reactions using 2‐aminobenzothiazole as a starting material in the synthesis of diverse heterocycles. These reactions enable the efficient and sustainable construction of novel bioactive molecules and pharmaceuticals. The current significance of these methodologies in modern organic chemistry is discussed, along with future perspectives. The review summarizes literature published from 2017 through early 2024.

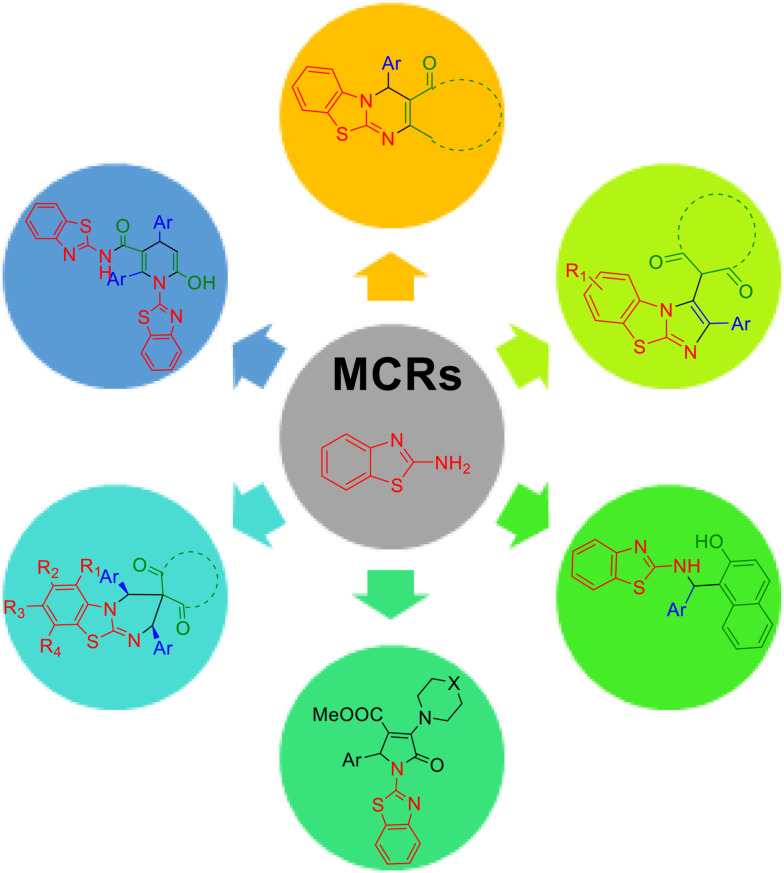

## 1 Introduction

Heterocycles are widely studied because they are present in various biological systems and have applications in medicine, agriculture, and materials science.[^1^, ^2^, ^3^, ^4^, ^5^, ^6^, ^7^, ^8^, ^9^, ^10^] One common method for constructing heterocycles is through multicomponent reactions (MCRs), where three or more reactants are simultaneously converted into the desired product. MCRs offer a powerful approach to combinatorial chemistry by allowing the synthesis of structurally diverse molecules from a limited set of simple starting materials. This process is performed in a single reaction vessel and often achieves high levels of efficiency and selectivity. However, designing effective and efficient MCR protocols remains challenging, particularly when sensitive functional groups are present in the starting materials.[^11^, ^12^, ^13^, ^14^, ^15^, ^16^, ^17^, ^18^]

The field of organic synthesis has made significant progress in developing efficient and modular methods for constructing complex molecules. Among the various building blocks that have emerged as versatile intermediates in such syntheses, 2‐aminobenzothiazole stands out due to its unique reactivity and ability to be incorporated into a wide range of heterocyclic systems. The use of 2‐aminobenzothiazole in MCRs has been particularly attractive due to its ability to participate in a variety of transformations, including condensation, cyclization, and heterocycle‐forming reactions. This has led to the development of efficient and modular methods for synthesizing heterocyclic compounds. These compounds are of significant interest due to their biological and medicinal properties.[^19^, ^20^, ^21^, ^22^]

2‐Aminobenzothiazole's versatility in MCRs is attributed to its ability to act as a nucleophile, an electrophile, or a Michael donor, depending on the reaction conditions and the nature of the other components involved. Recent advances in the application of 2‐aminobenzothiazole in the multicomponent synthesis of heterocycles have showcased its versatility in forming a variety of heterocyclic systems. These include isoxazoles, pyrazoles, imidazoles, pyridines, indoles, and fused heterocycles. The recent application of 2‐aminobenzothiazole to the multicomponent synthesis of heterocycles has led to the development of innovative strategies for rapidly assembling complex molecules. These strategies have potential applications in pharmaceuticals, biology, and materials science. The modular nature of these reactions allows for easy variation of the substituents on the 2‐aminobenzothiazole and other components, thus enabling the synthesis of large libraries of compounds for screening in various biological and material applications. This has created new avenues for rapidly constructing complex molecules with potential use as therapeutic agents, agrochemicals, and functional materials.[^23^, ^24^, ^25^, ^26^, ^27^, ^28^, ^29^, ^30^, ^31^, ^32^, ^33^, ^34^, ^35^, ^36^, ^37^]

This manuscript presents a detailed investigation of the application of 2‐aminobenzothiazole in the multicomponent synthesis of various heterocycles using MCR protocols,[^38^, ^39^, ^40^, ^41^, ^42^, ^43^] with the aim of elucidating the underlying mechanism(s) involved and identifying factors affecting the efficiency of the processes. The findings highlight the potential of 2‐aminobenzothiazole as a reliable and versatile synthon in MCRs, advancing the development of novel and sustainable methodologies for synthesizing complex heterocycles. The literature reported between 2017 and 2024 is analyzed.

## 2 The Chemistry of 2‐Aminobenzothiazole

**Figure 1:**
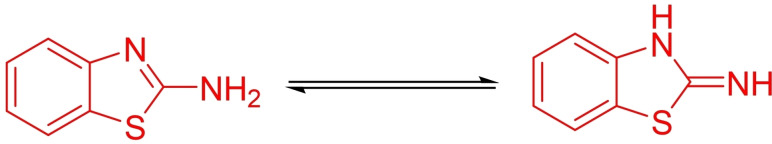
Structure of 2‐aminobenzothiazole.

The organic compound 2‐aminobenzothiazole has the formula C_7_H_6_N_2_S, and its structure can be expressed as a tautomerism of enamines (Figure 1). This tautomerism accounts for the reactivity and stability of the reagent. It is a heterocyclic aromatic amine that consists of a benzene ring fused to a thiazole group, which in turn is attached to an amino group. In this Section we will summarize some of the key points regarding the chemistry of 2‐aminobenzothiazole.

2‐Aminobenzothiazole's amino group gives it weakly basic properties. It can accept a proton (H^+^) to form an ammonium ion, which carries a positive charge. The compound exhibits aromaticity due to the delocalization of (п) electrons within both the benzene ring and the thiazole ring. This aromatic character greatly influences its reactivity in various chemical reactions.[^44^, ^45^, ^46^, ^47^]

2‐Aminobenzothiazole can undergo electrophilic aromatic substitution reactions, where an electrophile replaces a hydrogen atom on the aromatic ring. These reactions facilitate the molecule‘s functionalization and the introduction of different groups. The amino group in 2‐aminobenzothiazole can also act as a ligand in coordination chemistry, forming stable complexes with various metal ions. This interaction greatly affects the compound‘s properties and reactivity.[^48^, ^49^, ^50^, ^51^]

Chemists utilize 2‐aminobenzothiazole as a building block in the synthesis of more complex organic molecules. Its unique structural features make it a valuable starting material for the preparation of compounds with diverse properties. This versatile heterocyclic compound, with its combination of an amino group and a benzothiazole moiety, possesses a range of interesting properties that make it an attractive building block for the synthesis of complex molecules with potential biological and material applications. 2‐Aminobenzothiazole is a crucial intermediate in synthesizing various complex heterocyclic systems and other fused heterocyclic architectures. These include condensation, cyclization, and heterocycle formation, as either an electrophile or a nucleophile (Michael donor), depending on the reaction conditions and the nature of the other components involved. This multifaceted reactivity has enabled the synthesis of a broad range of heterocyclic compounds with potential applications in pharmaceuticals, agrochemicals, and materials science. Furthermore, it can undergo MCRs with aldehydes, ketones, nitroalkenes, 1,2‐diketones, and other partners to form a diverse array of heterocyclic compounds. These reactions offer a powerful strategy for rapidly assembling complex molecules from simple starting materials in a single operation.[^17^, ^18^, ^19^, ^20^, ^21^, ^22^, ^23^, ^24^, ^25^, ^26^, ^27^, ^28^, ^29^, ^30^, ^31^, ^32^, ^33^, ^34^, ^35^, ^36^, ^37^]

Researchers have explored the potential medicinal applications of derivatives of this compound. Compounds derived from 2‐aminobenzothiazole have been extensively studied for their diverse biological activities, which include range of compounds pertaining to antidepressant,^[52]^ human A3 receptor antagonists,^[53]^ anti‐Parkinson's,^[54]^ antitrypanocidal agents,^[55]^ antimalarial,^[56]^ anti‐microbial,[^57^, ^58^, ^59^, ^60^, ^61^] antidiabetic,^[62]^ antituberculosis,[^63^, ^64^, ^65^, ^66^, ^67^] β‐amyloid imagining agents,^[68]^ anti‐inflammatory,[^69^, ^70^, ^71^] apoptosis inducers,^[72]^ anti‐viral,[^73^, ^74^, ^75^] antifungal,^[76]^ anti‐tuberculosis,[^77^, ^78^] cytotoxic,[^79^, ^80^] anti‐urease agents,^[81]^ enzyme inhibitors,[^82^, ^83^] hemostatic agents,^[84]^ anti‐cancer,[^85^, ^86^, ^87^, ^88^] anti‐oxidant,^[89]^ anti‐ bacterial,[^90^, ^91^, ^92^] anthelmintic,^[93]^ muscle relaxants,^[94]^ and anticonvulsant[^90^, ^91^, ^92^] anthelmintic,^[93]^ muscle relaxants,^[94]^ and anticonvulsant properties.^[95]^

**Figure 2:**
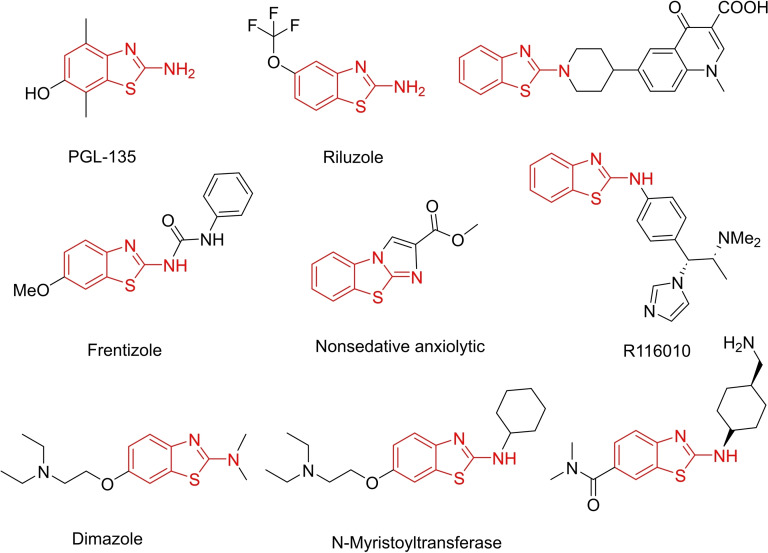
2‐Aminobenzothiazoles containing bioactive molecules.

These compounds are summarized in Figure 2.

In materials science, 2‐aminobenzothiazole and its derivatives have been utilized as building blocks for synthesizing functional materials, including organic semiconductors, light‐emitting diodes, and sensors. The tunable electronic properties of these compounds make them attractive candidates for applications in optoelectronics, sensing technologies, and electroluminescent devices.[^96^, ^97^, ^98^, ^99^] The versatility of this reaction makes it a powerful tool in several applications, such as vulcanization accelerators,^[100]^ plant growth regulators,^[101]^ imaging reagents,[^102^, ^103^, ^104^] fluorescence materials,[^105^, ^106^, ^107^] selective ion detectors,[^108^, ^109^, ^110^, ^111^] fumigants, and pest repellants.^[112]^

### 2.1 Classifying the Reactions of 2‐Aminobenzothiazole

**Figure 3:**
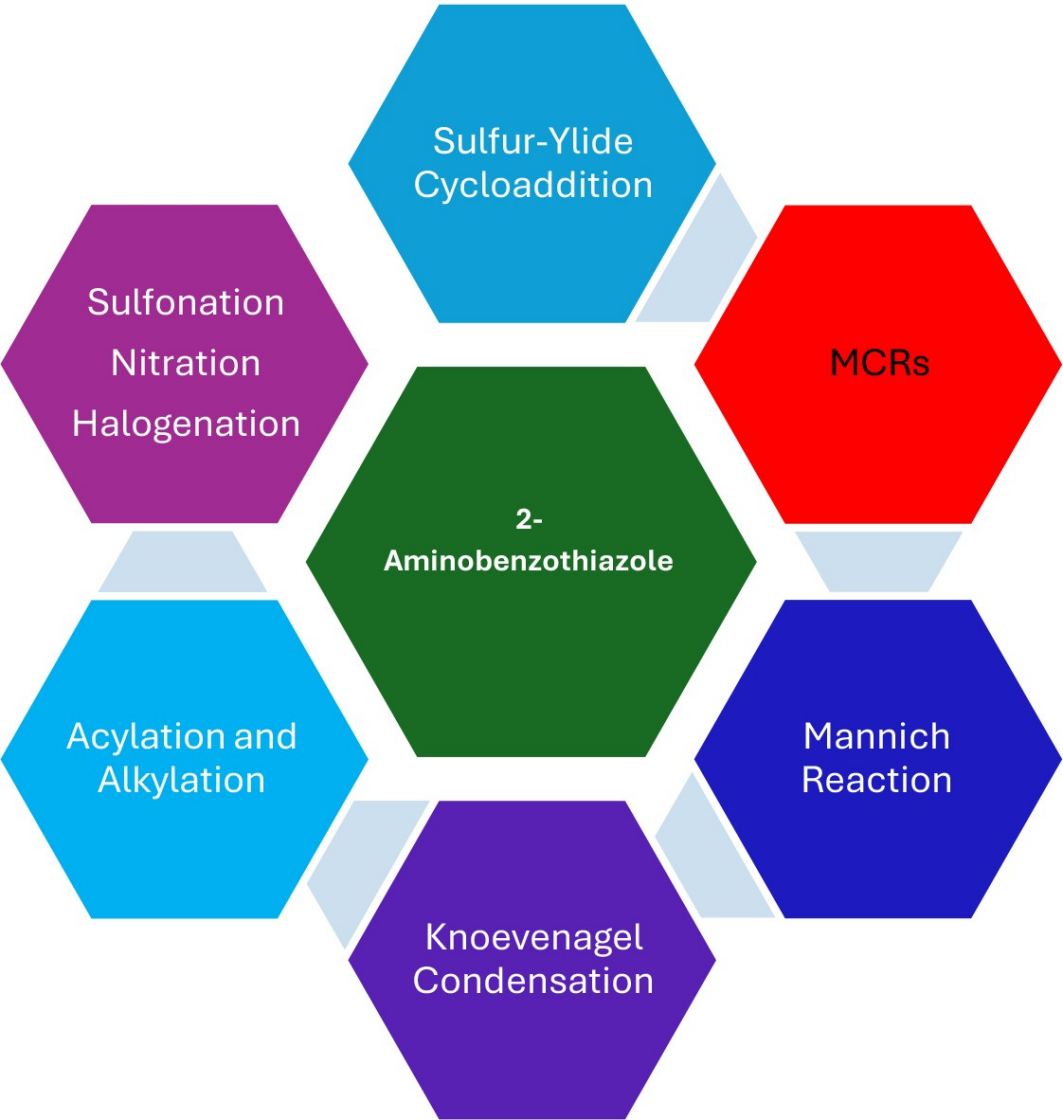
Reactions of 2‐aminobenzothiazole.

2‐Aminobenzothiazole is a versatile heterocyclic compound capable of participating in various chemical reactions due to the presence of the amino group and the benzothiazole ring.[^113^, ^114^, ^115^, ^116^, ^117^, ^118^] Some common reactions involving 2‐aminobenzothiazole are highlighted in Figure 3.

### 2.2 Synthesis of 2‐Aminobenzothiazoles

**Figure 4:**
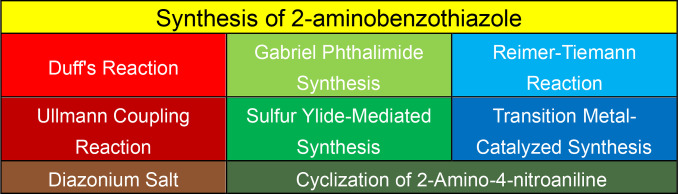
Synthesis of 2‐aminobenzothiazole.

The synthesis of 2‐aminobenzothiazole involves constructing the benzothiazole ring and subsequently introducing the amino group at the 2‐position. Several methods have been developed, each with its own advantages and limitations.[^113^, ^114^, ^115^, ^116^, ^117^, ^118^, ^119^, ^120^, ^121^, ^122^, ^123^, ^124^, ^125^, ^126^, ^127^, ^128^, ^129^] Some common methods for synthesizing 2‐aminobenzothiazole are shown in Figure 4.

The future of applying 2‐aminobenzothiazole to MCRs holds promise for advancing synthetic methodologies and accessing structurally diverse heterocyclic compounds.[^17^, ^18^, ^19^, ^20^, ^21^, ^22^, ^23^, ^24^, ^25^, ^26^, ^27^, ^28^, ^29^, ^30^, ^31^, ^32^, ^33^, ^34^, ^35^, ^36^, ^37^, ^38^, ^39^, ^40^, ^41^, ^42^, ^43^, ^44^, ^45^, ^46^, ^47^, ^48^, ^49^, ^50^, ^51^] Here are some key aspects highlighting the future applications of 2‐aminobenzothiazole in MCRs:

1. Diversity in heterocyclic compound synthesis: Using 2‐aminobenzothiazole in MCRs allows for the rapid and efficient construction of complex heterocyclic scaffolds in a single operation. This strategy is expected to lead to the discovery of novel heterocycles with diverse functionalities and potential applications in drug discovery, materials science, and other fields.
2. Green and sustainable synthesis: MCRs are environmentally friendly synthetic tools known for their atom‐ and step‐economy. Integrating 2‐aminobenzothiazole into MCRs aligns with the principles of green chemistry and offers a sustainable approach to accessing valuable heterocyclic compounds.
3. Drug discovery and development: 2‐aminobenzothiazole can serve as a key building block in MCRs, creating new avenues for synthesizing compound libraries with potential pharmacological activities. Its future application in MCRs can facilitate the rapid synthesis of diverse drug‐like molecules for screening and lead optimization in drug discovery programs.
4. Functional materials synthesis: The future application of 2‐aminobenzothiazole in MCRs extends beyond medicinal chemistry. It can lead to the synthesis of functional materials with tailored properties. Researchers can access new materials for applications in optoelectronics, catalysis, and more by incorporating 2‐aminobenzothiazole derivatives into MCRs.
5. Exploration of new reaction pathways: Ongoing research into the application of 2‐aminobenzothiazole in MCRs may unveil novel reaction pathways and transformations, expanding the synthetic toolbox for accessing diverse heterocyclic structures. This exploration could lead to the discovery of unprecedented chemical reactivity and innovative synthetic routes.

These heterocycles can be classified into six categories based on their structural characteristics: Pyrimidine frameworks, pyridine frameworks, benzimidazole frameworks, aminobenzothiazole frameworks, spiro frameworks, and miscellaneous compounds. In this article, we will discuss each of these categories in turn, specifically focusing on their formation *via* MCRs.

## 3 Pyrimidine Frameworks

**Scheme 1:**
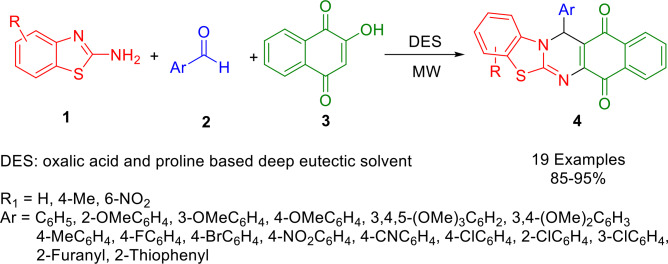
Synthesis of 13‐aryl‐13*H*‐benzo[*g*]benzothiazolo [2,3‐*b*]quinazoline‐5,14‐diones.

By reacting aromatic aldehydes (**2**), 2‐aminobenzothiazoles (**1**), and 2‐hydroxy‐1,4‐naphthoquinone (**3**) under microwave radiation, Ma *et al*. developed a three‐component method for synthesizing highly functionalized 13‐aryl‐13‐*H*‐benzothiazolo[2,3‐*b*]quinazoline‐5,14‐diones (**4**) that was both efficient and environmentally benign (Scheme 1). This reaction was conducted in a deep eutectic solvent (DES) composed of oxalic acid and proline, which functioned both as a catalyst and as an environment‐friendly reaction medium.^[130]^ The reported approach exhibits significant advantages, such as an easy work‐up, environment‐friendly reaction conditions, short reaction times, excellent yields, one‐pot MCR, chromatography‐free purification, and the reuseability of DES.^[131]^

**Scheme 2:**
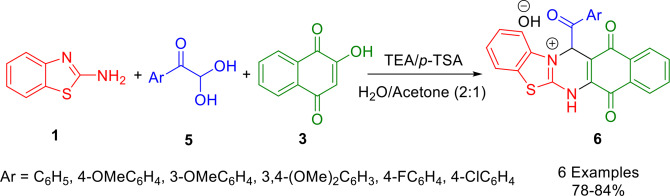
Synthesis of tetrahydrobenzo[*g*]benzo[4,5]thiazolo[2,3‐*b*]quinazolin‐14‐ium hydroxides.

Nouri *et al*. synthesized a new series of tetrahydrobenzo[*g*]benzo[4,5]thiazolo[2,3‐*b*]quinazolin‐14‐ium hydroxides **6** *via* a one‐pot, three‐component reaction of aryl glyoxal monohydrates (**5**), 2‐hydroxy‐1,4‐naphthoquinone (**3**), and 2‐aminobenzothiazoles (**1**) in the presence of triethylamine and *p*‐toluenesulfonic acid as organocatalysts in H_2_O/acetone (2 : 1) at room temperature (Scheme 2). This method offers mild reaction conditions, excellent yields, easy work‐up, operational simplicity, and readily accessible starting materials and catalysts. The synthesized new products may have valuable pharmaceutical and biological applications.^[132]^

**Scheme 3:**
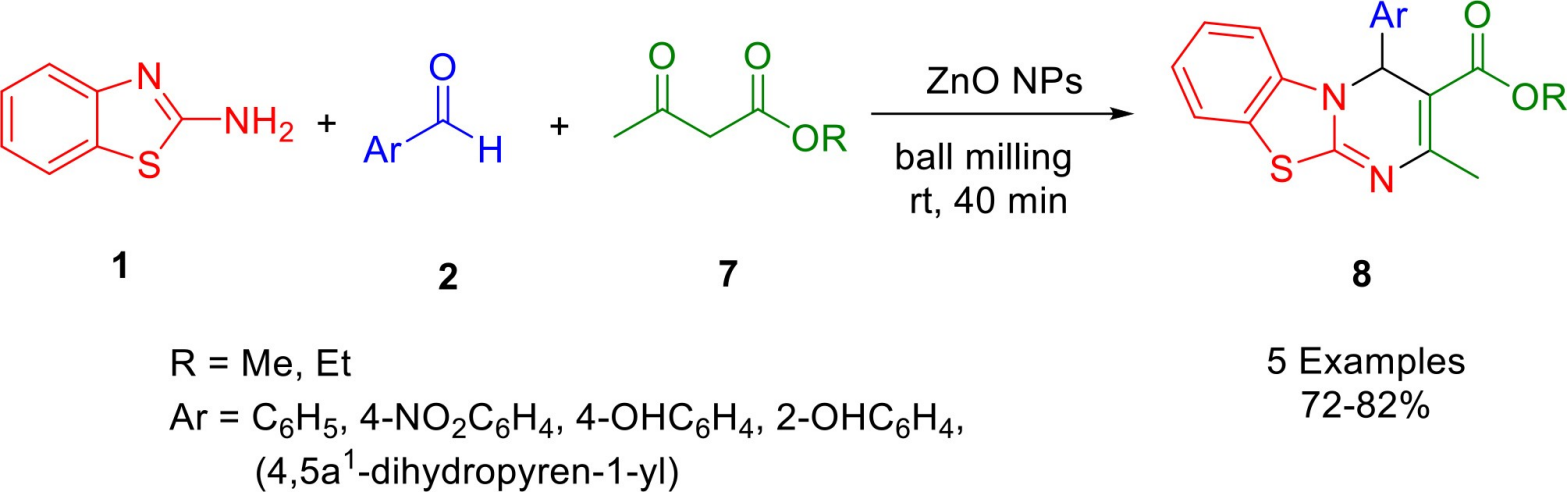
Synthesis of various pyrimidine derivatives.

By using a sol‐gel route, modified ZnO nanoparticles (NPs) were synthesized with an aromatic capping agent in order to provide a highly efficient catalyst. The study conducted by Jang *et al*. demonstrated the successful one‐pot synthesis of various functionalized pyrimidine derivatives (**8**) using a solvent‐free ball milling procedure (23) (Scheme 3). The starting materials for this reaction were 2‐aminobenzothiazole (**1**), substituted benzaldehydes (**2**), and ethyl acetoacetate (**7**). The mixture was milled with ZnO NPs in a tungsten carbide milling jar. It was possible to recycle catalyst NS‐5 up to five times without reducing its catalytic activity. A comparison of the current methodology with Ecoscale and E‐factor indicated that it provides a clean and green synthetic route for the synthesis of different pyrimidine derivatives. Additionally, the present method can be performed on a multigram scale, highlighting its potential for industrial application.^[133]^

**Scheme 4:**
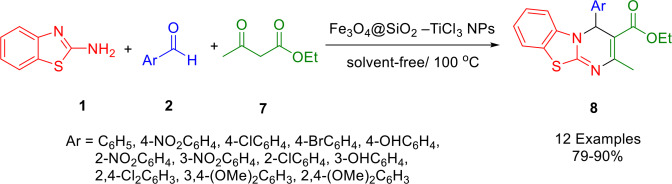
Synthesis of 4*H*‐pyrimido[2,1‐*b*]benzothiazole derivatives.

Using a magnetic catalyst under solvent‐free techniques at 100 °C, Fazeli‐Attar *et al*. performed a three‐component, domino‐type reaction for the regioselective synthesis of functionalized 4*H*‐pyrimido[2,1‐*b*]benzothiazole derivatives with a short reaction time (Scheme 4). This method provided high yields of functionalized naphthyridine derivatives **37** starting from 2‐aminobenzothiazole (**1**), aldehydes (**2**), ad ethylacetoacetate. An ecofriendly heterogeneous catalyst composed of Fe_3_O_4_@SiO_2_‐TiCl_3_ nanoparticles was prepared by the magnetization of Fe_3_O_4_@SiO_2_ followed by treatment with titanium tetrachloride (TiCl_4_). Catalysts should ideally be reusable and this catalyst could be reused four times without losing significant catalytic activity. High yields and purity of the synthesized compounds were obtained following a short reaction time.^[134]^

**Scheme 5:**
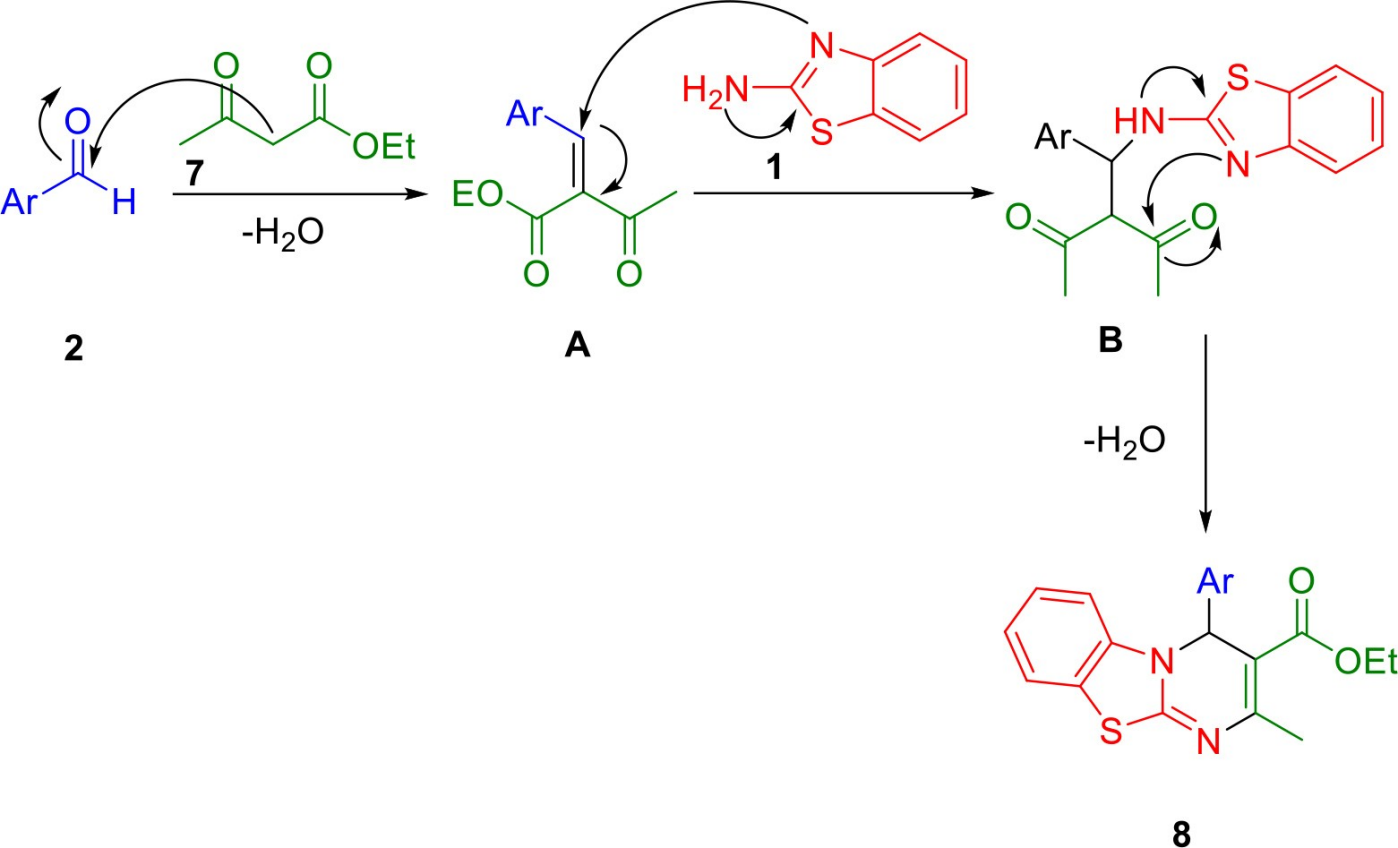
A proposed mechanism for preparation of 4*H*‐pyrimido[2,1‐*b*]benzothiazole derivatives.

A conceivable pathway for the reaction between ethylacetoacetate (**7**), an aldehyde (**2**), and 2‐aminobenzothiazole (**1**) is depicted in Scheme 5. Initially, the aldehyde′s and β‐ketoester′s carbonyl groups are attached to and activated by Ti present in the catalyst, preparing them for subsequent condensation reactions. The condensation between the activated aldehyde and β‐ketoester occurred *via* a Knoevenagel reaction, resulting in the formation of compound (**A**). Subsequently, 2‐aminobenzothiazole (**1**) undergoes a Michael addition with compound (**A**), leading to the creation of an iminium ion (**B**). Following a proton shift and cyclization process, the compounds 4*H*‐pyrimido[2,1‐*b*]benzothiazole derivatives are synthesized.

**Scheme 6:**
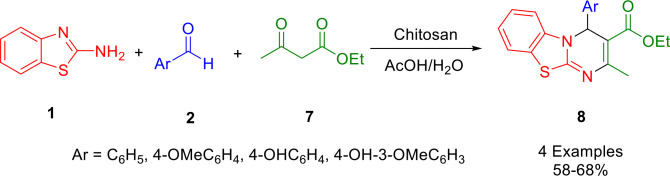
Synthetic procedure for 4*H*‐benzo[4,5]thiazolo[3,2‐*a*]pyrimidine‐3‐carboxylates.

By combining benzaldehyde derivatives (**2**), ethyl acetoacetate (**7**), and 2‐aminbenzothiazole (**1**) in the presence of chitosan dissolved in refluxing acetic acid, Verma and colleagues synthesized a panel of 4*H*‐benzo[4,5]thiazolo[3,2‐*a*]pyrimidine‐3‐carboxylate derivatives (**8**) (Scheme 6). In this study, four heterocyclic compounds containing nitrogen, sulfur, and oxygen atoms were investigated for their ability to inhibit mild steel corrosion in HCl. The ability to resist corrosion was dependent on the nature of the substituents present on the molecular structures. Molecular dynamics simulations have been conducted to ascertain the molecular alignment of the inhibitors on the metallic surface. The results indicate that the inhibitors align horizontally on the metallic surface to offer the most effective protection. The polarization study demonstrated that cathodic inhibitors primarily impeded electron flow. A strong correlation was observed between the computational and experimental results.^[135]^

**Scheme 7:**
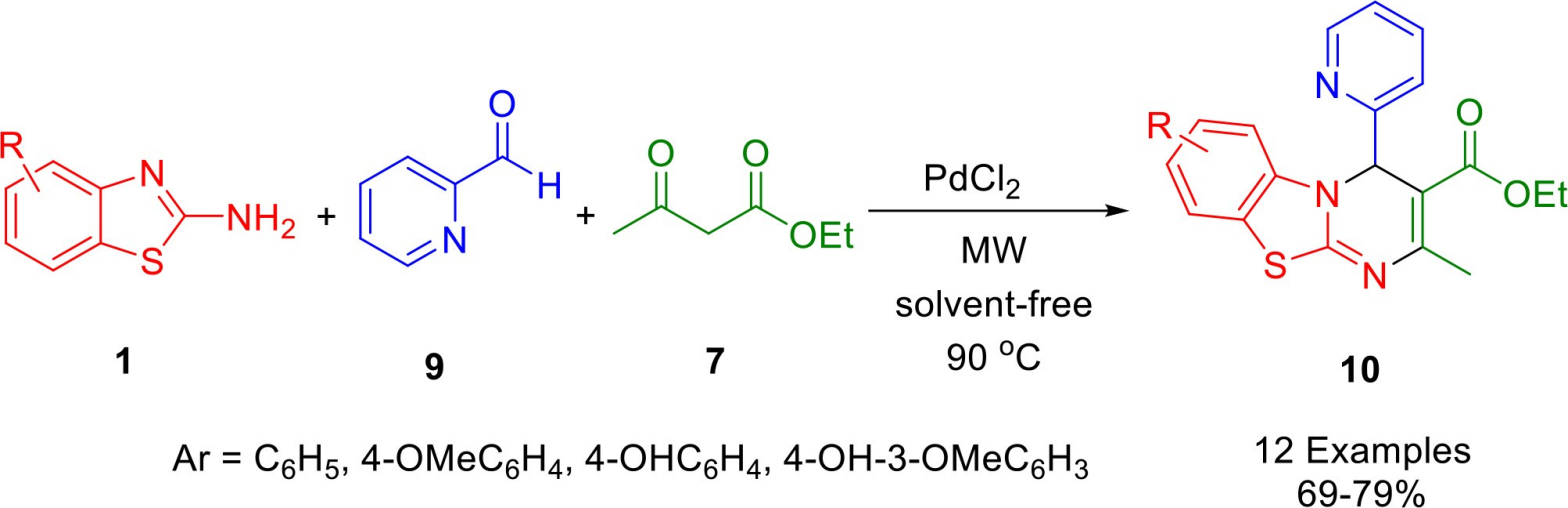
Synthesis of 4*H*‐pyrimido[2,1‐*b*]benzothiazoles derivatives.

The microwave‐assisted synthesis of ethyl 2‐methyl‐4‐(pyridin‐2‐yl)‐4*H*‐benzo[4,5]thiazolo[3,2‐*a*]pyrimidine‐3‐carboxylate derivatives (**10**) described by Patel and co‐workers was achieved by reacting 2‐aminobenzothiazole derivatives (**1**) with pyridine 2‐aldehyde (**9**) and ethyl acetoacetate (**7**) under solvent free conditions, using PdCl_2_ as a catalyst (Scheme 7). Among its salient characteristics are its mild reaction conditions, ease of setup, simplicity, convergence, high atom economy, short reaction time, and environmental friendliness. The structure of compound (**10**) was confirmed by X‐ray analysis of a single crystal.^[136]^

They conducted the reactions under solvent‐free conditions, meaning that the reactions were significantly safer, less toxic, and more environmentally friendly. Furthermore, the presence of three different heterocyclic motifs, such as benzothiazole, pyridine, and pyrimidine in a single molecule makes these derivatives very valuable in biological and pharmacological applications. *In vitro* tests were performed on the synthesized compounds to determine their antibacterial activity against two gram‐negative and two gram‐positive strains of bacteria. Antioxidant activity was also evaluated as by means of the DPPH radical scavenging assay. Antioxidant antibacterial activity of moderate to good was observed for all compounds. The compounds (R = Me), and (R = NO_2_) showed very good inhibition against *M. tuberculosis* H37RV. The other compounds showed moderate to good activity.

**Scheme 8:**
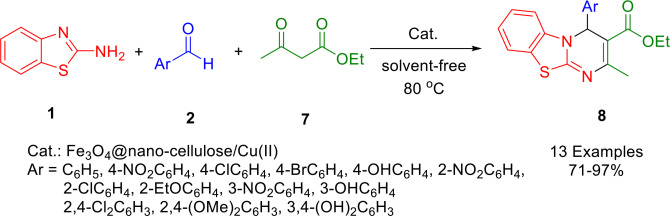
Synthesis of 4*H*‐pyrimido[2,1‐*b*]benzothiazole derivatives.

In a three‐component reaction involving aromatic aldehydes (**2**), 2‐aminobenzothiazole (**1**), and ethyl acetoacetate (**7**), Mirjalili and colleagues synthesized 4*H*‐pyrimido[2,1‐*b*]benzothiazoles (**8**) by using Fe_3_O_4_@nanocellulose/Cu(II), a novel magnetite recoverable, environmentally friendly, inexpensive, and efficient nanocatalyst at 80 °C under solvent‐free conditions (Scheme 8). To prepare a green bio‐based magnet, aqueous suspensions of nano‐cellulose were coprecipitated with Fe^2+^ and Fe^3+^ ions *in situ*. In order to perform recycling experiments, a magnet was used to separate the catalyst, which was then washed with ethanol and dried at room temperature. In an oven at 80 °C, the catalyst was washed twice with ethanol and water. Good yields, environmental friendliness, facile setup, and moderate reusability were some of the benefits of this protocol.^[137]^

**Scheme 9:**
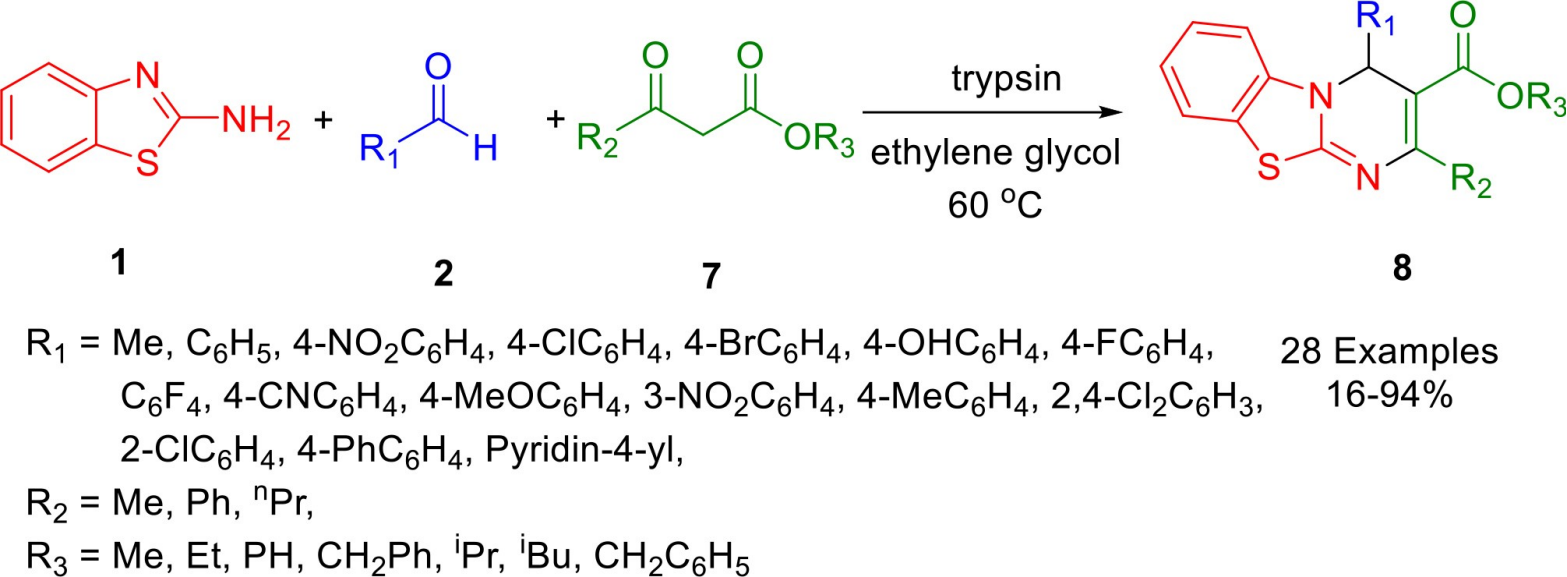
Synthesis of 4*H*‐pyrimido[2,1‐*b*]benzothiazoles.

In comparison with traditional chemo‐catalysts, enzymes provide more efficient and eco‐friendly ways to construct various functional molecules. Wang and co‐workers synthesized 4*H*‐pyrimido[2,1‐*b*] benzothiazole derivatives (**8**) by performing a Biginelli reaction involving aldehydes (**2**), ketoesters (**7**) and 2‐aminobenzothiazole (**1**) in one pot. The use of trypsin provided a streamlined pathway for producing different ring‐fused pyrimidines at 60 °C in ethylene glycol (Scheme 9). The range of substrates could be further extended to include acetaldehyde, a chemical liquid with a relatively low boiling point and usually difficult to handle above room temperature, as well as commercially available aromatic aldehydes. This was achieved by using vinyl acetate as an *in‐situ* acetaldehyde generator. Almost all the substrates tested demonstrated satisfactory reactivity. AIE (Aggregation‐Induced Emission) was also observed in several substrates, which have been investigated as biomarkers. Cellular experiments indicate that these heterocyclic fluorophores may be able to be used for biosensors and organelle targeting.^[138]^

**Scheme 10:**
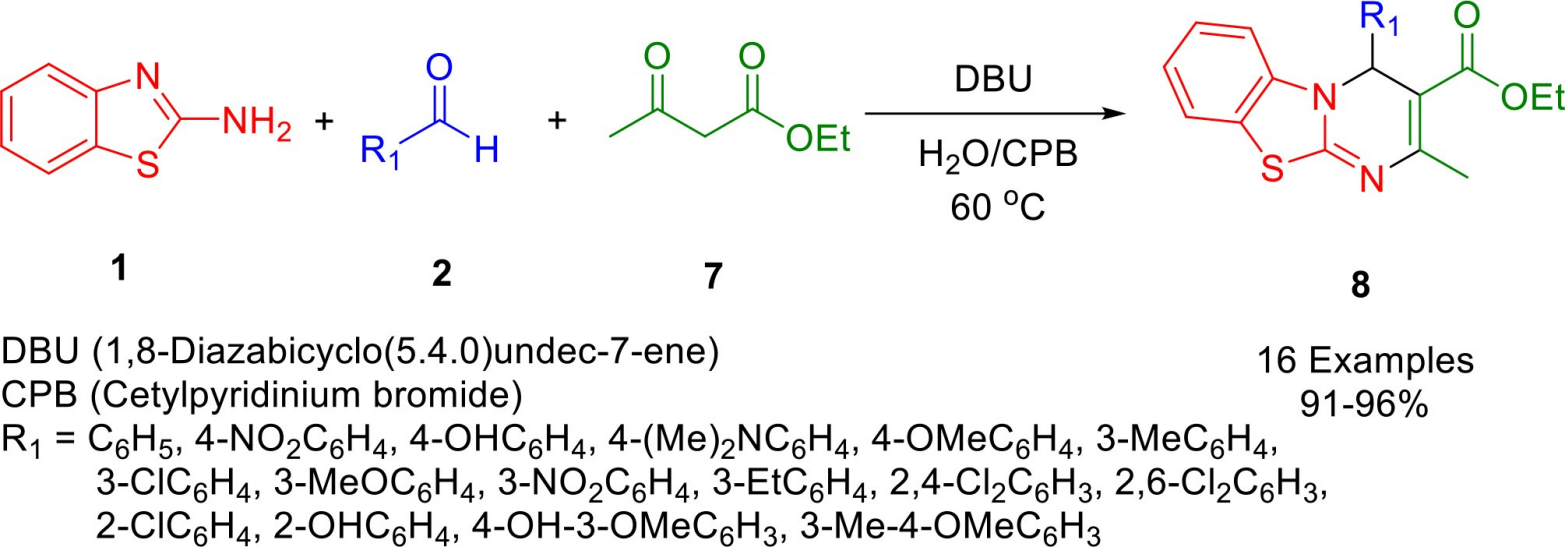
Synthesis of tricyclic 4*H*‐pyrimido[2,1‐*b*]benzothiazoles derivatives.

A simple, practical, and scalable method was described for synthesizing triheterocyclic 4*H*‐pyrimido[2,1‐*b*]benzothiazole derivatives (**8**). Yields in the range of 91–96 % could be achieved by cyclo‐condensing ketoester (**7**) in a micellar medium with substituted aromatic aldehydes (**2**) and 2‐aminobenzothiazole (**1**) in one pot (Scheme 10). Catalyzed by 1,8‐Diazabicyclo(5.4.0)undec‐7ene (DBU) as an organic base catalyst in micellar media and cetylpyridinium bromide (CPB) surfactant in aqueous medium, the reaction is green, cheap, and sustainable. The products formed in a short reaction time in good to excellent yields. Synthesis on a multigram scale was also demonstrated. In addition to being environmentally benign, metal‐free, easy to work‐up, and using no hazardous solvents, the developed methodology has the advantage of having high atom economy and low E‐factor in comparison to related protocols, alongside the ability to purify the products without column chromatography.^[139]^

**Scheme 11:**
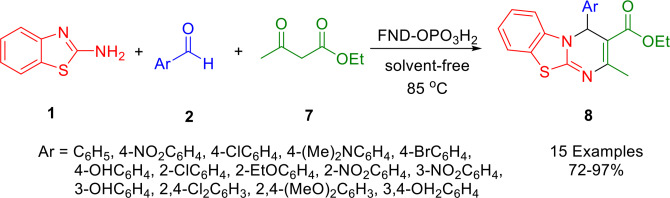
Synthesis of 4*H*‐pyrimido[2,1‐*b*]benzothiazoles.

Using a one‐pot three‐component reaction with ethyl acetoacetate (**7**), 2‐aminobenzothiazole (**1**), and a variety of aryl aldehydes (**2**) under solvent‐free conditions at 85 °C, using Fe_3_O_4_@nano‐dextrin‐OPO_3_H_2_ as a nanocomposite catalyst, Mirjalili and his colleagues synthesized 4*H*‐pyrimido[2,1‐*b*]benzothiazole derivatives (**8**) in 72–97 % yields (Scheme 11). This was successfully performed using Fe_3_O_4_@nano‐dextrin as a magnetic core‐shell structure, which was treated with P_4_O_10_ to yield Fe_3_O_4_@nano‐dextrin‐OPO_3_H_2_ as a bio‐based nano‐catalyst. The heterogeneous catalyst shows high efficiency, and can be reused for subsequent reactions after being washed three times with ethanol and dried. The methodology is eco‐friendly, providing good yields of products that are easy to purify.^[140]^

**Scheme 12:**
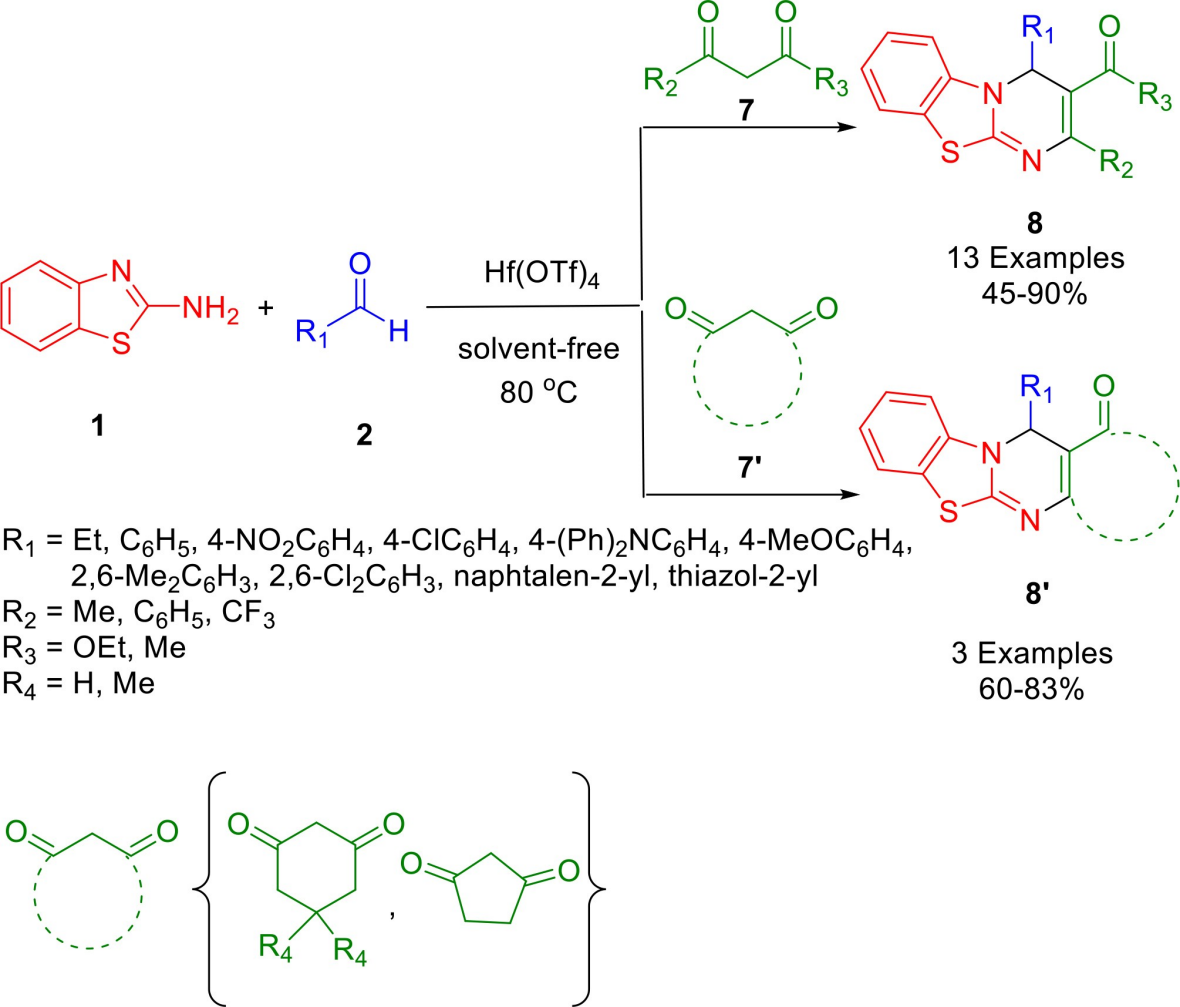
Synthesis of pyrimido[2,1‐*b*][1,3]benzothiazole (PBT) scaffolds.

In 2021, with the help of a Hf(OTf)_4_‐catalyzed one‐pot strategy, Gong and colleagues developed a route to pyrimido[2,1‐*b*][1,3]benzothiazole (PBT)‐based aggregation‐induced emission luminogens (AIEgens). The AIEgens synthesized were fully color tunable, yielding solid state fluorescence quantum yields of up to 86.8 %. The compounds were also crystallizable. Solvent‐free methods were used to yield novel PBT derivatives (**8** and **8’**) from a diverse panel of aldehydes (**2**), diketones (**7** and **7’**), 2‐aminobenzothiazoles (**1**), and amines (Scheme 12). The energy of the excited state of PBT AIEgens is dissipated by rotation of the exocyclic carbonyl (ester/ketone) as an atypical propeller‐shaped molecule, rather than through the phenyl group (as is more commonly observed). PBT AIEgens have unique structure–property relationships and an AIE mechanism that can be analyzed theoretically and crystallographically. AIEgens with a PBT core structure demonstrate quantum yields up to 86.8 %, with the solid‐state fluorescence emissions of the current compound library spanning the entire visible spectrum.^[141]^

**Scheme 13:**
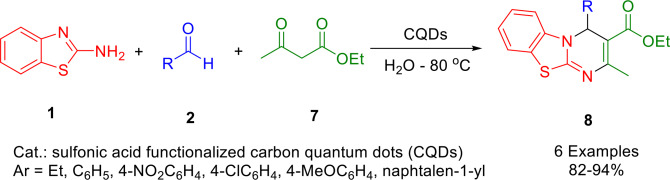
Synthesis of pyrimidine derivatives.

Based on a literature review, carbon quantum dots (CQDs) have been extensively studied as an alternative to conventional nanomaterials in catalysis. Singh *et al*. developed an efficient, metal‐free, green, and heterogeneous catalyst derived from biomass that is based on carbon quantum dots (CQDs) functionalized with sulfonic acid (Scheme 13). For the synthesis of benzothiazole‐pyrimidine derivatives (**8**) using benzaldehyde (**2**), ethyl acetoacetate (**7**), and 2‐aminobenzothiazole (**1**), they employed a one‐pot multicomponent strategy using condensation reactions. Product yields ranged from moderate to high in all cases. Furthermore, the catalyst is capable of being reused up to six times without significantly degrading its catalytic performance. A simple and efficient aqueous synthetic protocol using an efficient biomass‐derived catalyst has therefore been developed to allow the facile synthesis of biologically important heterocycles. As far as green chemistry protocols are concerned, the developed strategy provides good eco‐scale and E‐factor values.^[142]^

**Scheme 14:**
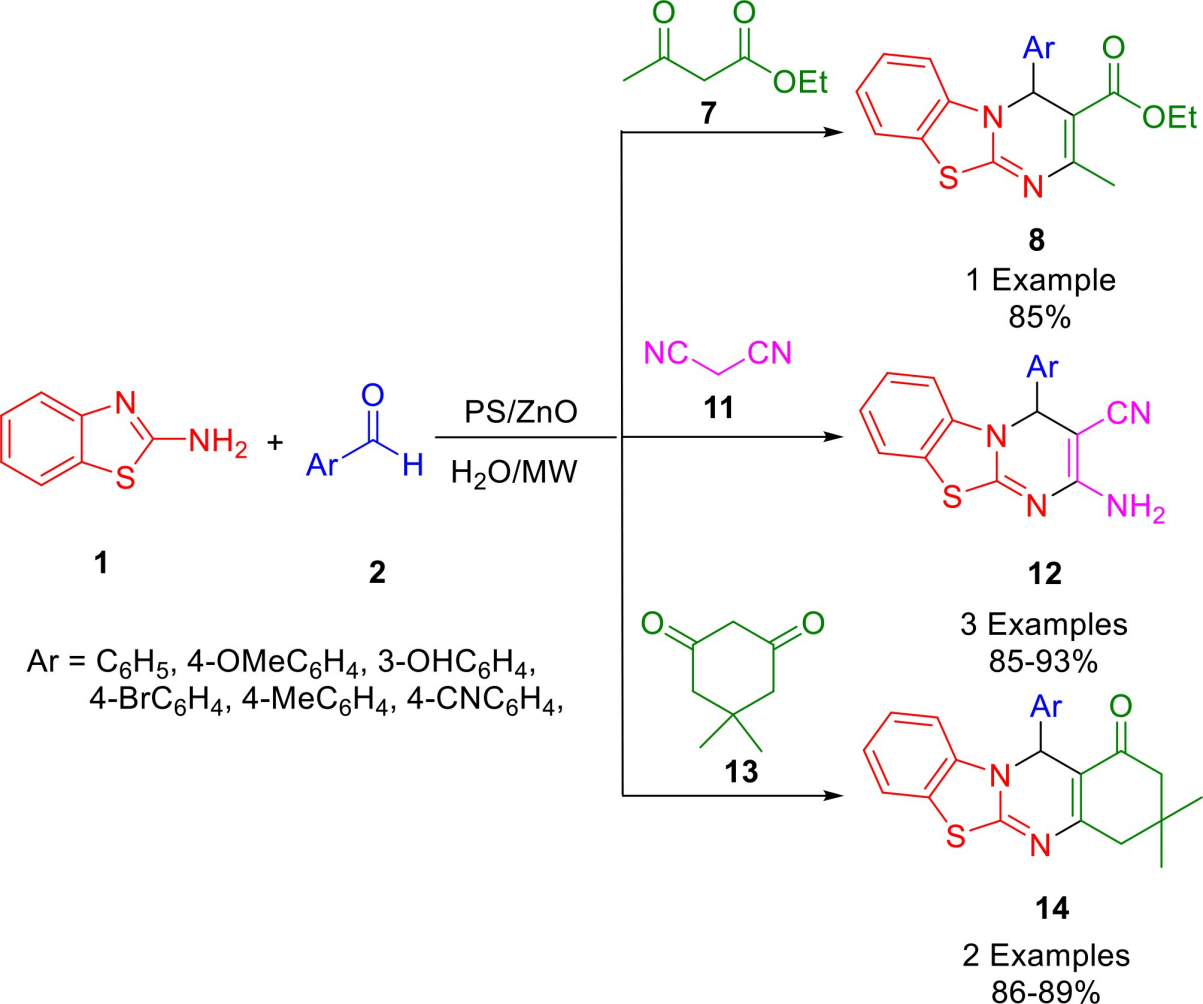
Synthesis of pyrimido[1,2‐*b*]benzazole derivatives.

Using aromatic aldehydes (**2**), 2‐aminobenzothiazoles (**1**), and C−H acidic compounds, Javanshir and coworkers were able to construct densely substituted pyrimido[1,2‐*b*]benzazole derivatives (**8**, **12** and **14**). The use of dimedone (**13**), malononitrile (**11**), or ethyl acetoacetate (**7**) in water under microwave irradiation, using a new bio nanocomposite (BNC) as a heterogeneous catalyst, delivered the desired products in high yields (Scheme 14). Using lignocellulosic waste peanut shells (LCWPS) as the raw material, a new BNC PS/ZnO was synthesized *in situ* using hydrothermal synthesis. LCWPS, a bio‐sourced polymeric carbohydrate, was studied for the benefit of sustainable management.^[143]^

**Scheme 15:**
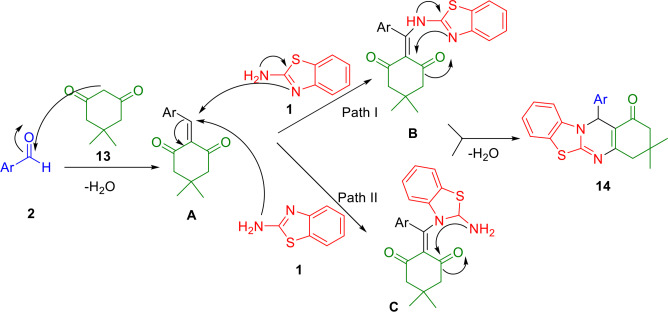
Suggested mechanism for the model reaction.

A plausible mechanism for the model reaction is shown in Scheme 15. PS/ZnO has been found to act as a Lewis acid/base catalyst in this mechanism. The Knoevenagel reaction between activated aldehyde (**2**) and dimedone (**13**) forms intermediate **A**. This is followed by a Michael addition of 2‐aminobenzothiazole (**1**) onto intermediate **A**, which can follow two possible mechanistic pathways (I or II), depending on whether the 2‐aminobenzimidazole (**1**) is attacked by the nitrogen in the side chain amine or the nitrogen in the ring. Pathway I results in the formation of intermediate B, while pathway II results in the formation of intermediate C. A final condensation reaction produces the required pyrimido[1,2‐*b*]benzazole derivatives (**14**).

**Scheme 16:**
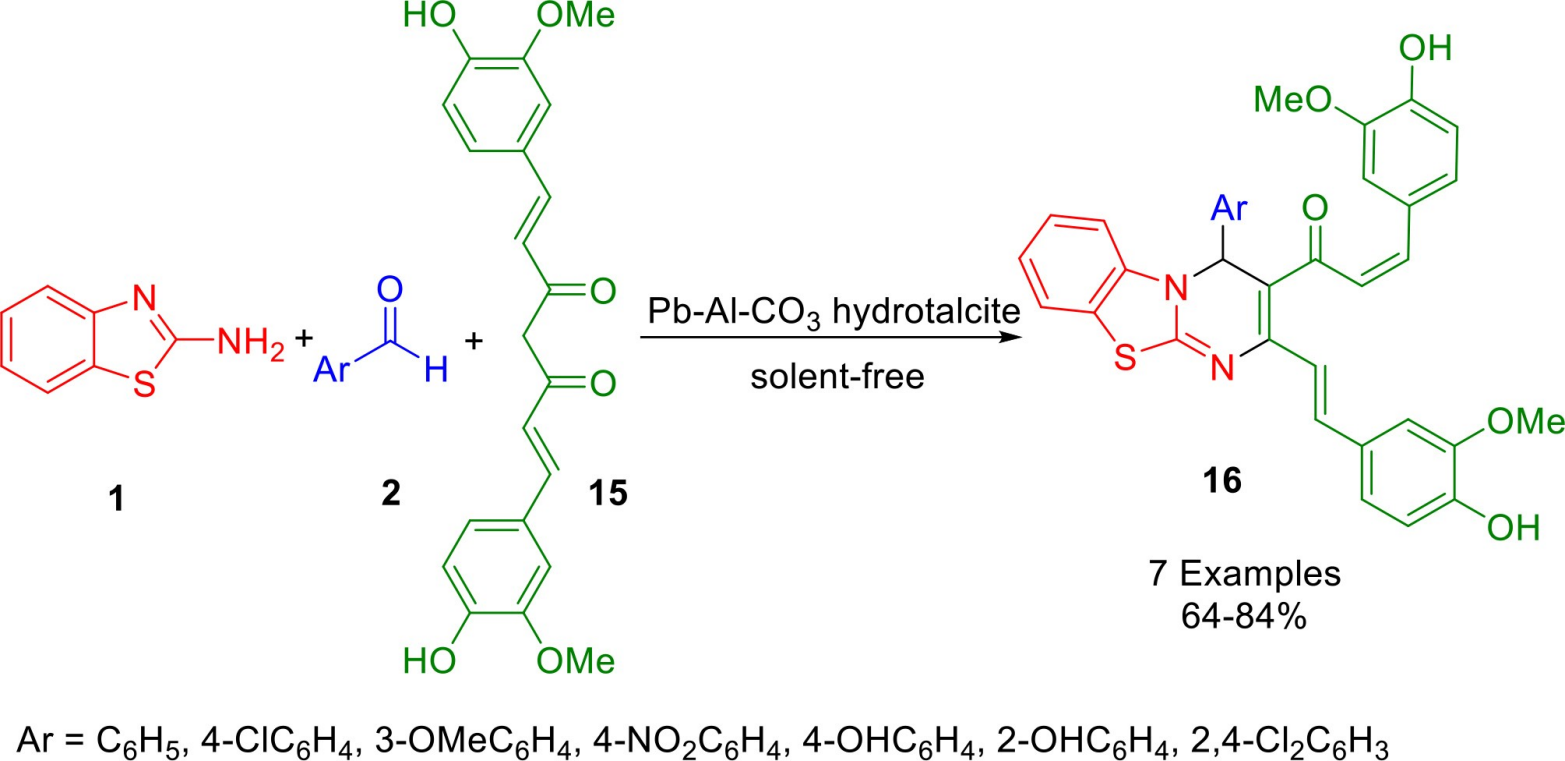
Synthesis of 4*H*‐pyrimido[2,1‐*b*]benzothiazole derivatives.

Al−Pb‐CO_3_ hydrotalcite was employed by Singh *et al*. as an inexpensive heterogeneous catalyst to synthesize functionalized 4*H*‐pyrimido[2,1‐*b*]benzothiazole derivatives (**16**) of curcumin (Scheme 16). Using this hydrotalcite catalyst, they developed a very clean and cost‐effective method for synthesizing 4‐phenyl‐4*H*‐pyrimido[2,1‐*b*][1,3]benzothiazolecurcumin (**16**), by combining substituted aromatic aldehydes (**2**), curcumin (**15**), and 2‐aminobenzothiazole (**1**) under solvent‐free conditions. The hydrotalcite provides a short reaction time, while being non‐toxic, reusable, and requiring minimal processing. As a result, this process is environmentally friendly. In addition to their antibacterial, anti‐inflammatory, antimicrobial, and cancer‐preventive properties, 4*H*‐pyrimido[2,1‐*b*]benzothiazole derivatives (**16**) of curcumin may also have significant antitumor properties.^[144]^

**Scheme 17:**
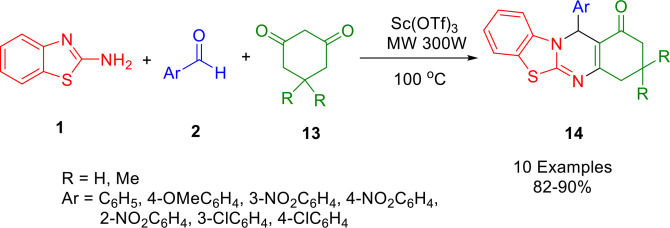
Synthesis of benzothiazolo quinazolinones.

Using microwave‐assisted synthesis, Gajaganti *et al*. constructed benzothiazolo quinazolinone derivatives (**14**) quickly and efficiently, in an environmentally friendly manner (**14**) (Scheme 17). Under solvent‐free conditions, the reaction of 2‐aminobenzothiazole (**1**), aromatic aldehydes (**2**), and 1,3‐diketones (**13**) produced excellent yields of the desired derivatives. The combination of microwave irradiation and scandium triflate was optimal for this method, offering many benefits such as excellent yields, minimal reaction time, and easy product isolation.^[145]^

**Scheme 18:**
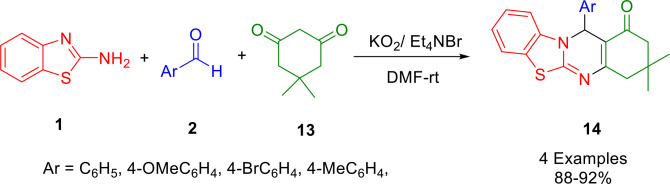
Synthesis of tetraheterocyclicbenzothiazolo quinazolin‐1‐ones.

Kumari and co‐workers reported a convenient one‐pot multicomponent synthesis of tetraheterocyclicbenzothiazoloquinazolin‐1‐one derivatives (**14**) under non‐aqueous conditions (Scheme 18), in the presence of tetraethylammonium superoxide. The mild reaction conditions conferred by superoxide produced benzothiazolo quinazolin‐1‐one derivatives at room temperature from various aromatic aldehydes (**2**), 2‐aminobenzothiazole (**1**), and dimedone (**13**). In a dry DMF solution, potassium superoxide and tetraethylammonium bromide participated in a phase transfer reaction to yield tetraethylammonium superoxide. Several structurally diverse, drug‐like, and complex heterocycles (quinazolines) were synthesized by phase transfer using tetraethylammonium bromide as the phase transfer catalyst.^[146]^

**Scheme 19:**
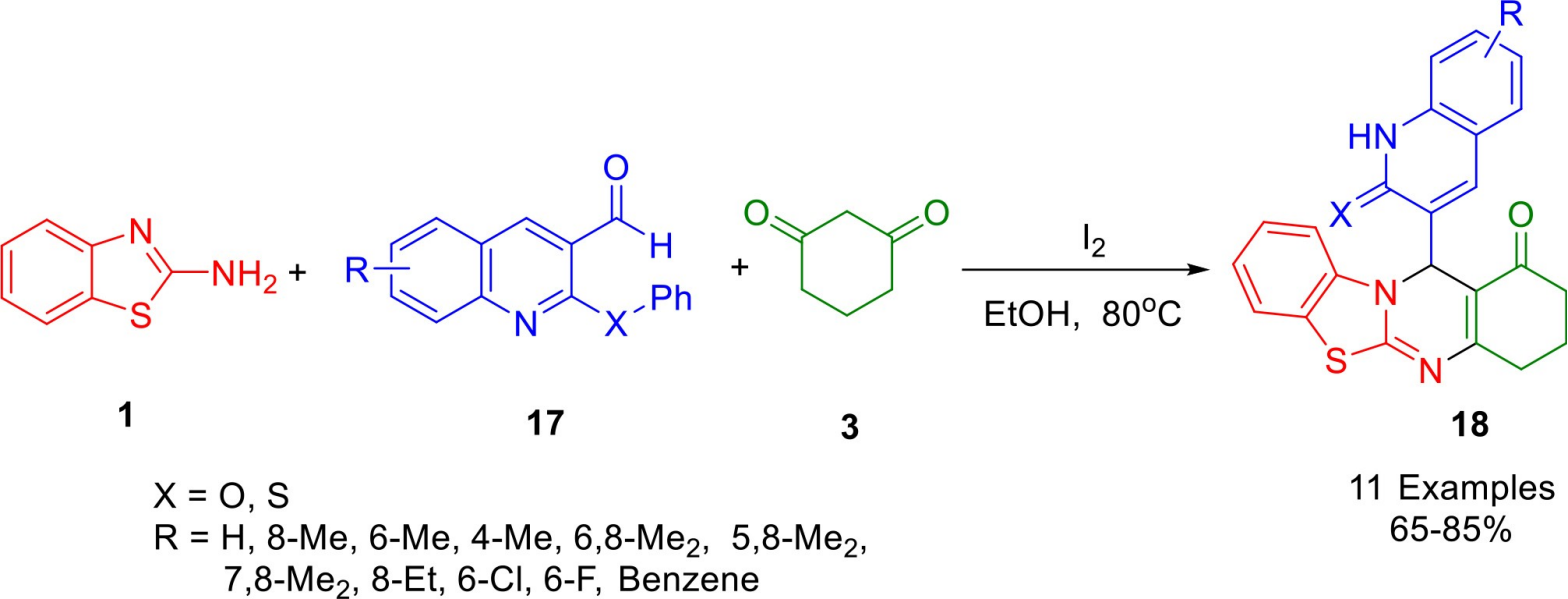
Synthesis of tetrahydro‐1*H*‐benzo[4,5]thiazolo [2,3‐*b*]quinazolin‐1‐one.

The tetrahydro‐1*H*‐benzo[4,5]thiazolin‐1‐one compounds (**18**) exhibit remarkable medicinal properties through various derivatives. As such, a three‐component one‐pot synthesis using a cyclo‐condensation reaction was developed to produce a fused molecule containing three heterocyclic units. According to Manne *et al*., the multicomponent reaction was carried out with substituted‐2‐phenoxyquinoline‐3‐carbaldehyde/substituted‐2‐phenylthioquinoline‐3‐carbaldehyde (**17**), 2‐aminobenzothiazole (**1**) and 1,3‐cyclohexanedione (**13′**) at 80 °C in ethanol, using iodine as a catalyst, producing tetrahydro‐1*H*‐benzo[4,5]thiazolin‐1‐one derivatives (**18**) in good to high yields (Scheme 19). There are several benefits to the present methodology, including ecologically friendly synthetic routes, excellent yields, mild reaction conditions, inexpensive catalysts, short reaction times, and very simple setup procedures.^[147]^

**Scheme 20:**
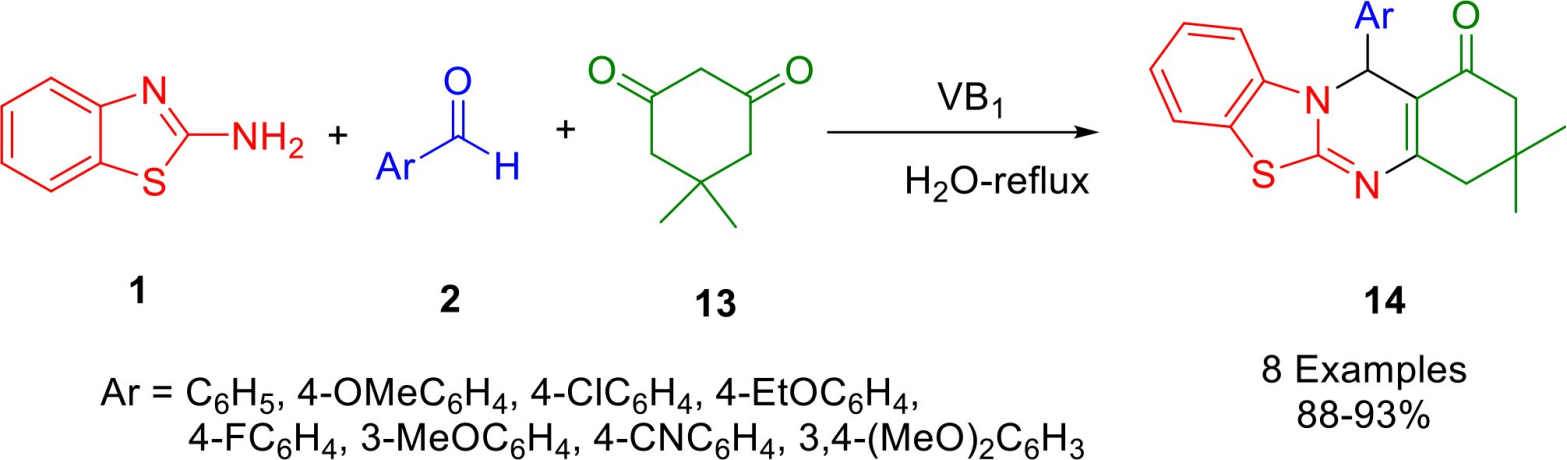
Synthesis of 4*H*‐pyrimido[2,1‐*b*]benzothiazoles.

By using a one‐pot, multicomponent reaction of aromatic aldehydes (**2**), 2‐aminobenzothiazole (**1**), and dimedone (**13**) in the presence of thiamine hydrochloride (VB1) as a biodegradable and reusable organocatalyst in water under reflux conditions, Sethiya *et al*. described a practical and rapid method for synthesis of novel pyrimidine derivatives (**14**), with yields ranging from 84 to 92 % (Scheme 20). By using VB_1_ as a catalyst, metal contamination was eradicated from the products, making it viable for eco‐friendly pathways and an improved catalyst for sustainable conditions. In addition to these fascinating features, the protocol was designed to include high product yields, milder reaction conditions, the avoidance of toxic solvents, and the easy recovery and reuse of the catalyst. In order to dock the synthesized compounds with DNA gyrase (1KZN) and dihydropteroate synthase (saDHPS) from *Staphylococcus aureus*, 6CLV and 6CLV were synthesized. Furthermore, the ligands′ ability to bind to the proteins showed some promise. As compared to the other synthesized compounds, compound (Ar=4‐CNC_6_H_4_) showed the best binding interactions as well as the best docking scores against 1KZN and 6CLV.^[148]^

**Scheme 21:**
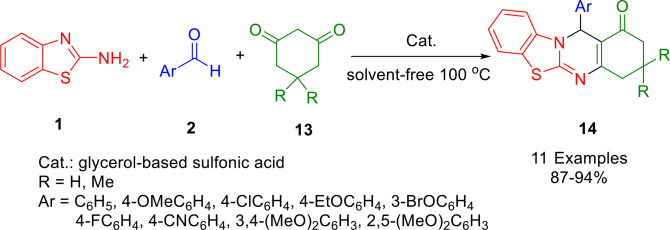
Synthesis of quinazoline analogs.

In 2022, Agarwal and co‐workers synthesized several novel fused quinazoline hybrids using a straightforward multicomponent cascade strategy. With a catalytic amount of glycerol‐based sulfonic acid, aromatic aldehydes (**2**), 1,3‐cyclic diketones (dimedone (**13**)/cyclohexanedione) and 2‐aminobenzothiazole (**1**), the desired fused quinazoline hybrids (**14**) were formed in 80–94 % yields (Scheme 21). Metal contamination is eliminated through the use of glycerol‐based sulfonic acid as a catalyst. According to this MCR strategy, Knoevenagel condensation is followed by Michael addition in a sequence similar to that of the Biginelli reaction. The present protocol proved to have a high tolerance for functional groups through a series of fused derivatives. In addition to the high yields of desired products, the design of the pathway is environmentally friendly, solvent‐free, and utilizes a heterogeneous catalyst that can easily be recovered and reused. Furthermore, the antimicrobial results showed that some of the compounds were more active than ciprofloxacin in preventing the growth of bacteria. The compounds (Ar=4‐FC_6_H_4,_ R= H) and (Ar=4‐CNC_6_H_4,_ R= H) were found to be more effective than fluconazole against the fungal strains tested.^[149]^

**Scheme 22:**
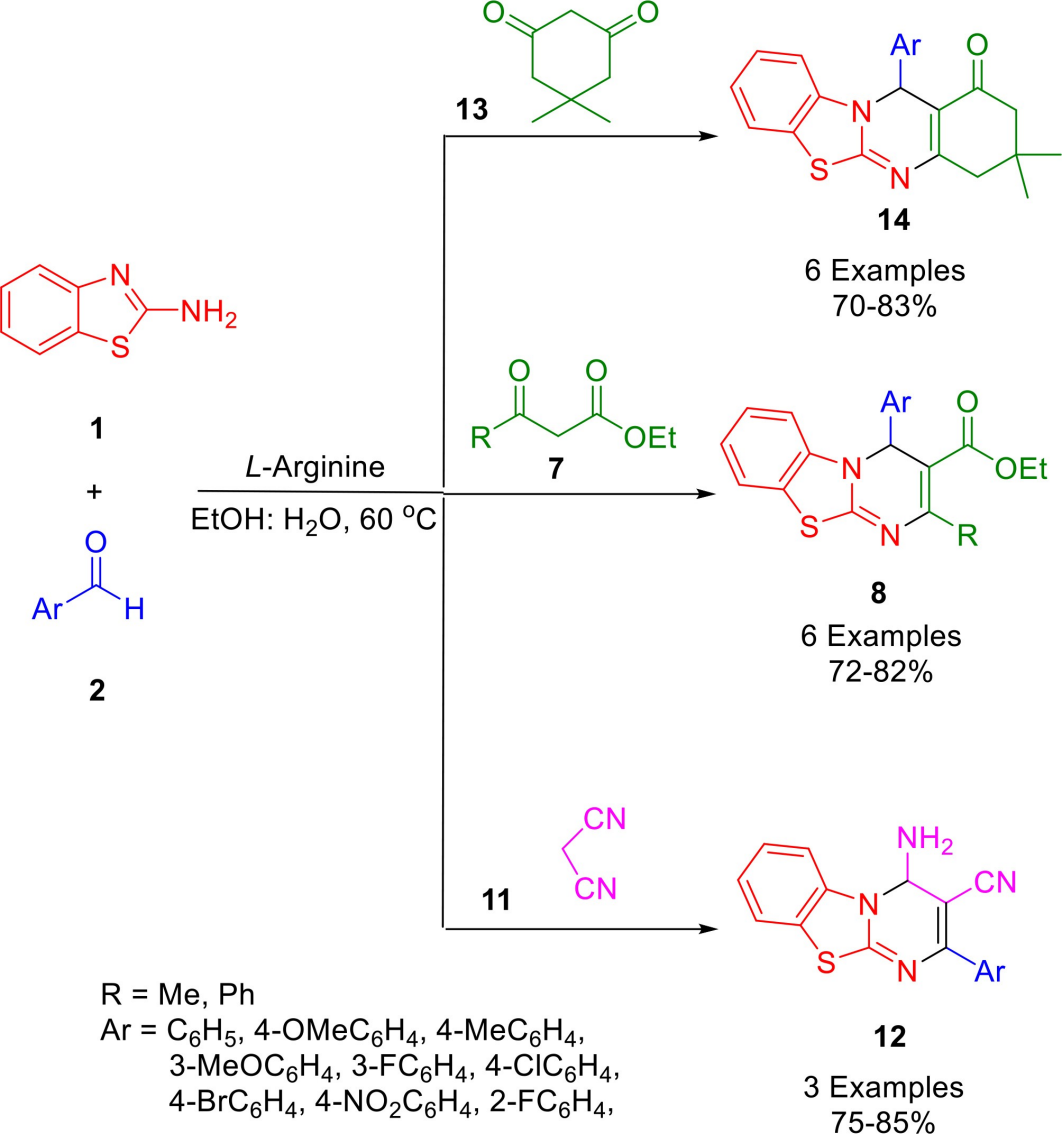
Synthesis of thiazolo[3,2‐*a*]pyrimidine.

In 2023, Singh and his research group developed a highly efficient and straightforward L‐arginine catalyzed approach for the synthesis of a variety of thiazolo[3,2‐*a*]pyrimidines (**14**, **8**, **12**) (Scheme 22). By using L‐arginine is an easily available, biodegradable, inexpensive, promising bio‐organic molecule as the catalyst, and EtOH: H_2_O (1 : 1) as a solvent system, the one‐pot pseudo‐three‐component reaction of aromatic aldehydes (**2**) and 2‐aminobenzothiaozole (**1**), with malononitrile (**11**), dimedone (**13**), or ethyl acetoacetate (**7**) afforded the corresponding thiazolo[3,2‐*a*]pyrimidines (**14**, **8**, **12**) in 70–85 % yield. The recycled promoter could be reused four times without any perceivable loss of activity. The recyclability of the catalyst negates the disadvantage of the high catalyst loading needed for this reaction. The major advantages of the present methodology, such as recyclability of catalyst, operational simplicity, easy scale‐up, wide substrate scope, easy work‐up, low cost, excellent yields, and high atom economy, make it a distinct improvement over the prevailing strategies.^[150]^

**Scheme 23:**
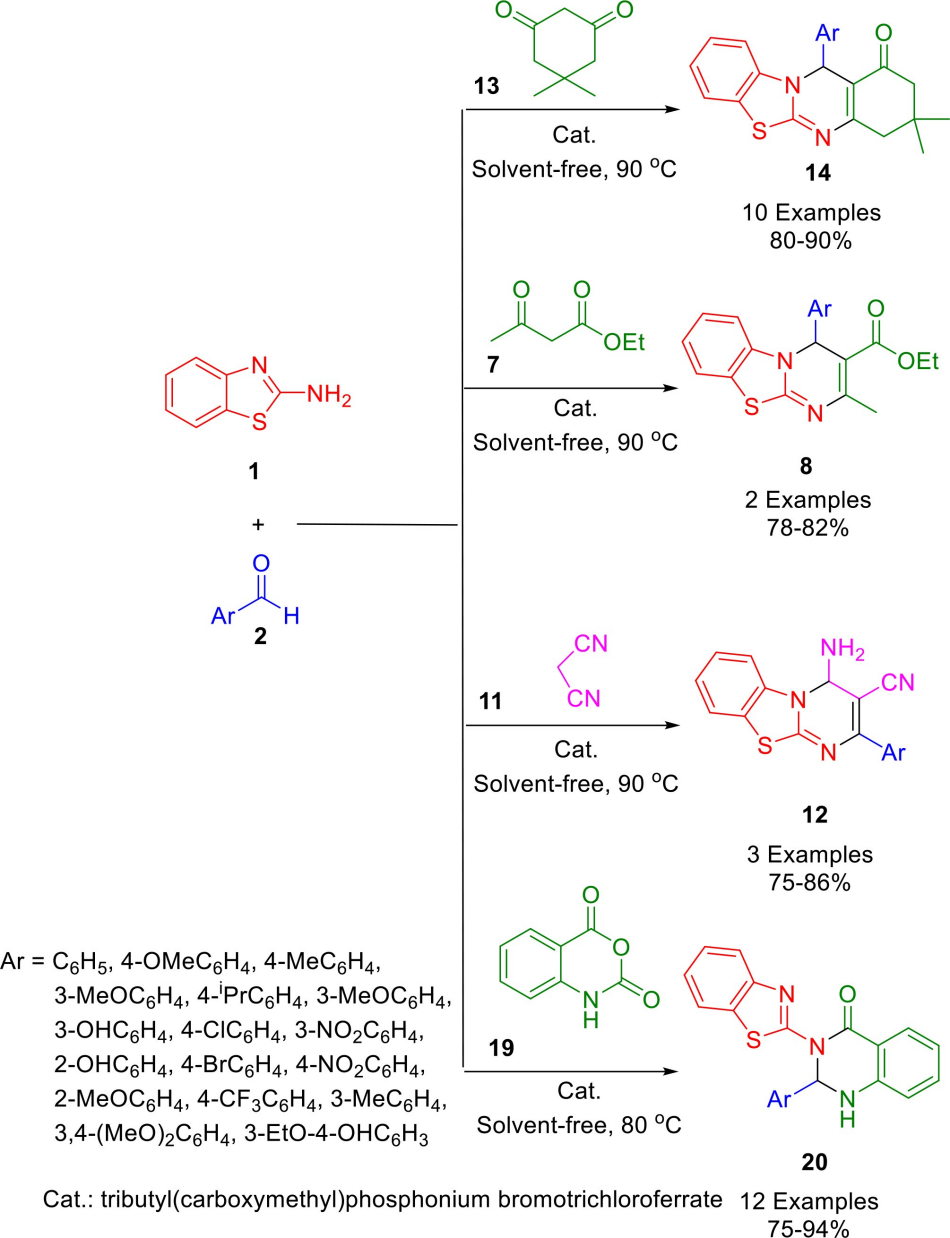
Synthesis of 2,3‐dihydroquinazolin‐4(1*H*)‐one derivatives and benzothiazolo[2,3‐*b*]quinazolin‐1‐one derivatives.

In a one‐pot, three‐component reaction of aldehyde derivatives (**2**), 2‐aminobenzothiazole (**1**), isotoeic anhydride (**19**), and a dicarbonyl compound (dimedone (**13**), ethyl acetoacetate (**7**), or malononitrile (**11**)), Zolfigol prepared 2,3‐dihy‐droquinazolin‐4(1*H*)‐ones and 4*H*‐pyrimidobenzothiazole derivatives in good to high yields (Scheme 23). As a heterogeneously catalyzed reaction, 10 mol% of magnetic ionic liquid (II) was used in an oil bath at 80 or 90 °C under solvent‐free conditions. Several new magnetic ionic liquids with acetic acid tags have been synthesized here, including tributyl(carboxymethyl)phosphonium bromotrichloroferrate. For the synthesis of the target molecules, the present strategy offers several advantages, such as high yields, short reaction times, low catalyst loadings, and safe and mild experimental protocols.^[151]^

**Scheme 24:**
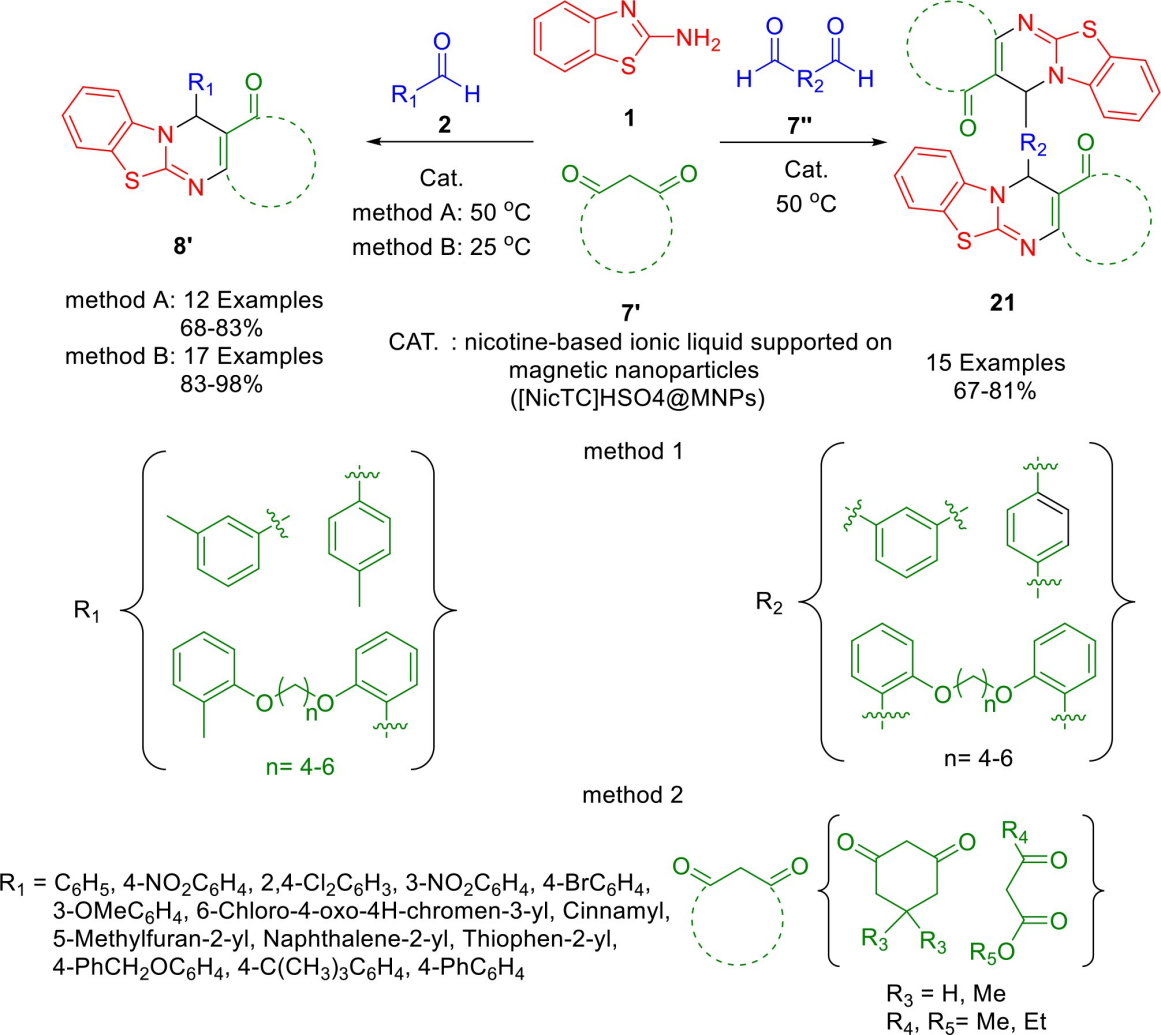
Synthesis of mono‐ and bis‐4*H*‐pyrimido[2,1‐*b*]benzothiazoles.

Alishahi *et al*. reported a new nicotine‐based organocatalyst supported on silica nanoparticles (Fe(III)‐NicTC@nSiO_2_) as a reusable heterogeneous catalyst for the regioselective formation of a series of novel mono‐ and bis‐4*H*‐pyrimido[2,1‐*b*]benzothiazole derivatives (**8′**, **21**). This reaction involves a three‐component between 2‐aminobenzothiazole (**1**), aldehydes (**2**)/dialdehydes (**7′′**), and *β*‐ketoesters/1,3‐diketones (**7′**) under solvent‐free conditions (Scheme 24). In this study, an acidic nicotine‐based ionic liquid supported on magnetic nanoparticles ([NicTC]HSO_4_@MNPs) was synthesized and characterized by different techniques. Furthermore, the catalyst exhibits mild reaction conditions, high yields, excellent selectivity, and facile recovery and reusability. It can be reused at least five times without any significant loss of its efficiency and activity. Conversely, this method can be made to be economically and environmentally benign.^[152]^

**Scheme 25:**
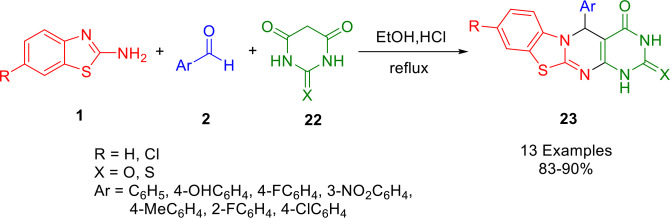
Synthesis of pyrimido[4,5‐*d*]pyrimidine derivatives.

A multicomponent reaction was utilized by Bodke and co‐workers to synthesize substituted phenyl‐1,5‐dihydro‐2*H*‐benzo[4,5]thiazolo[3,2‐*a*]pyrimido[4,5‐d]pyrimidine derivatives (**23**) (Scheme 25). A reaction between 2‐aminobenzothiazole (**1**), barbituric/thiobarbituric acid (**22**), and substituted benzaldehydes (**2**) in ethanol using hydrochloric acid as a catalyst under reflux conditions was used to produce the desired product. The compounds were tested for antimicrobial and antifungal activity on a variety of microbial strains such as *E. coli*, *P. Syringae*, *S. Aureus*, *F. oxysporum*, *A. favus* and *A. solani*. The tests indicated that compound (R=H, X=S, Ar=C_6_H_5_) and compound R=H, X=O, Ar=C_6_H_5_) were the most effective against all the pathogens tested. A molecular docking study was also conducted on selected compounds *in silico*.^[153]^

**Scheme 26:**
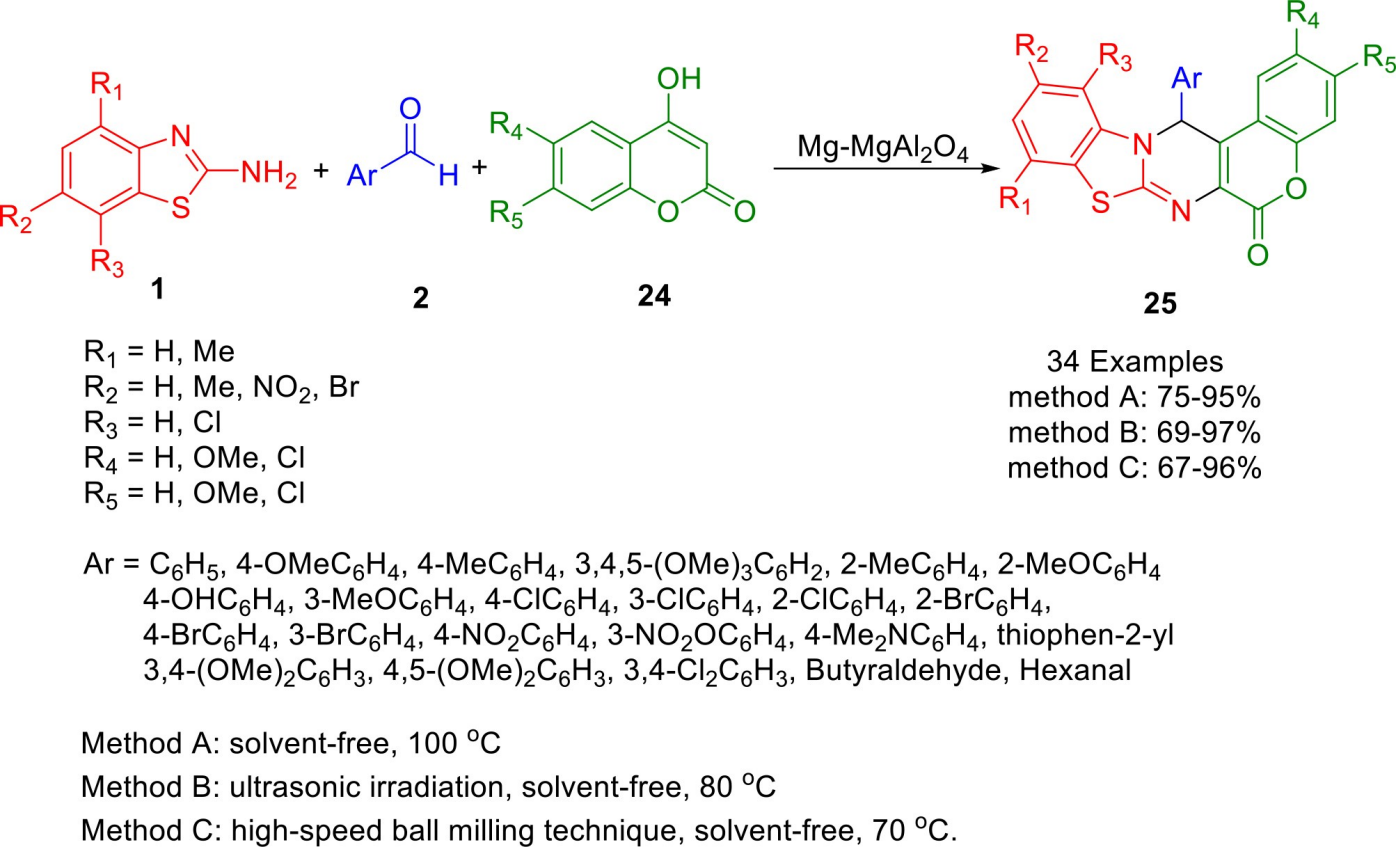
Synthesis of benzo[4,5]thiazolo[3,2‐*a*]chromeno[4,3‐*d*]pyrimidin‐6‐one derivatives.

By reacting 4‐hydroxycoumarins (**24**) with aldehydes (**2**) and 2‐aminobenzothiazole derivatives (**1**) under solvent‐free conditions in the presence of MgO‐MgAl_2_O_4_ nanocomposite as a recyclable catalyst, Khalaj synthesized benzo[4,5]thiazolo[3,2‐*a*]chromene[4,3‐*d*]pyrimidin‐6‐one derivatives (**25**) in good to excellent yields (Scheme 26). In this experiment, Mg(NO_3_)_2_ and Al(NO_3_)_3_ salts were co‐precipitated to form MgO and MgAl_2_O_4_. Using thermal, solvent free, ultrasonic irradiation (US) and high‐speed ball milling (HSBM), the researchers investigated the catalytic activity of MgO‐MgAl_2_O_4_ nanocomposite, as well as the synthesis of the above‐mentioned compounds.^[154]^

**Scheme 27:**
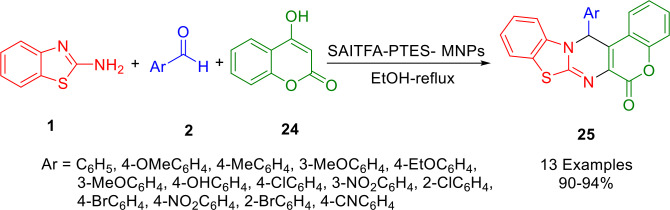
Synthesis of fused pyrimidine derivatives.

In 2020, Basirat synthesized fused pyrimidine derivatives (**25**) from 4‐hydroxycoumarin (**24**), benzaldehydes (**2**) and aminobenzothiazole (**1**) in ethanol. Yields of 90–94 % were obtained with the help of a TFA‐SAI‐PTES‐MNPs nanocatalyst (Scheme 27). The innovative nanocatalyst was synthesized by supporting 3‐(3‐sulfamic acid imidazolium trifluoroacetate)propyl triethoxysilane on magnetic nanoparticles. In these one‐pot, multi‐component reactions under thermal conditions, magnetic catalysts were investigated for their catalytic activity. With high levels of catalytic activity and short reaction times, this catalyst demonstrated excellent catalytic properties and was easily separated using an external magnet. The catalyst was reusable four times without losing significant catalytic efficiency.^[155]^

**Scheme 28:**
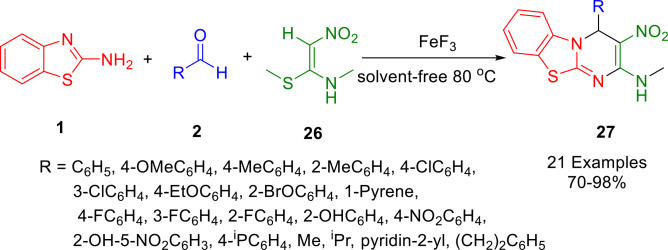
Synthesis of N‐methyl‐3‐nitro‐4*H*‐pyrimido[2,1‐*b*] benzothiazole‐2‐amine derivatives.

An effective one‐pot condensation of 2‐aminobenzothiazole (**1**), 1‐(methylthio)‐2‐nitroethenamine (**26**), and aldehydes (**2**) was reported in 2019 by Atar *et al*. Under neat conditions (Scheme 28), a multi‐component strategy was used to construct a library of functionalized *N*‐methyl‐3‐nitro‐4*H*‐pyrimido [2,1‐*b*] [1,3] benzothiazole‐2‐amine derivatives (**27**) with excellent yields. There are no adverse effects on the yield of the products from recovering and reusing the catalyst, FeF_3_. Using this green protocol, *N*‐methyl‐3‐nitro‐4*H*‐pyrimido [2,1‐*b*] [1,3‐*a*] benzothiazole‐2‐amine is produced in a one‐pot process. The results show that the catalyst has excellent recyclability and reusability, as the catalyst was able to be reused four times without suffering a loss of catalytic activity.^[156]^

## 4 Pyridine Frameworks

**Scheme 29:**
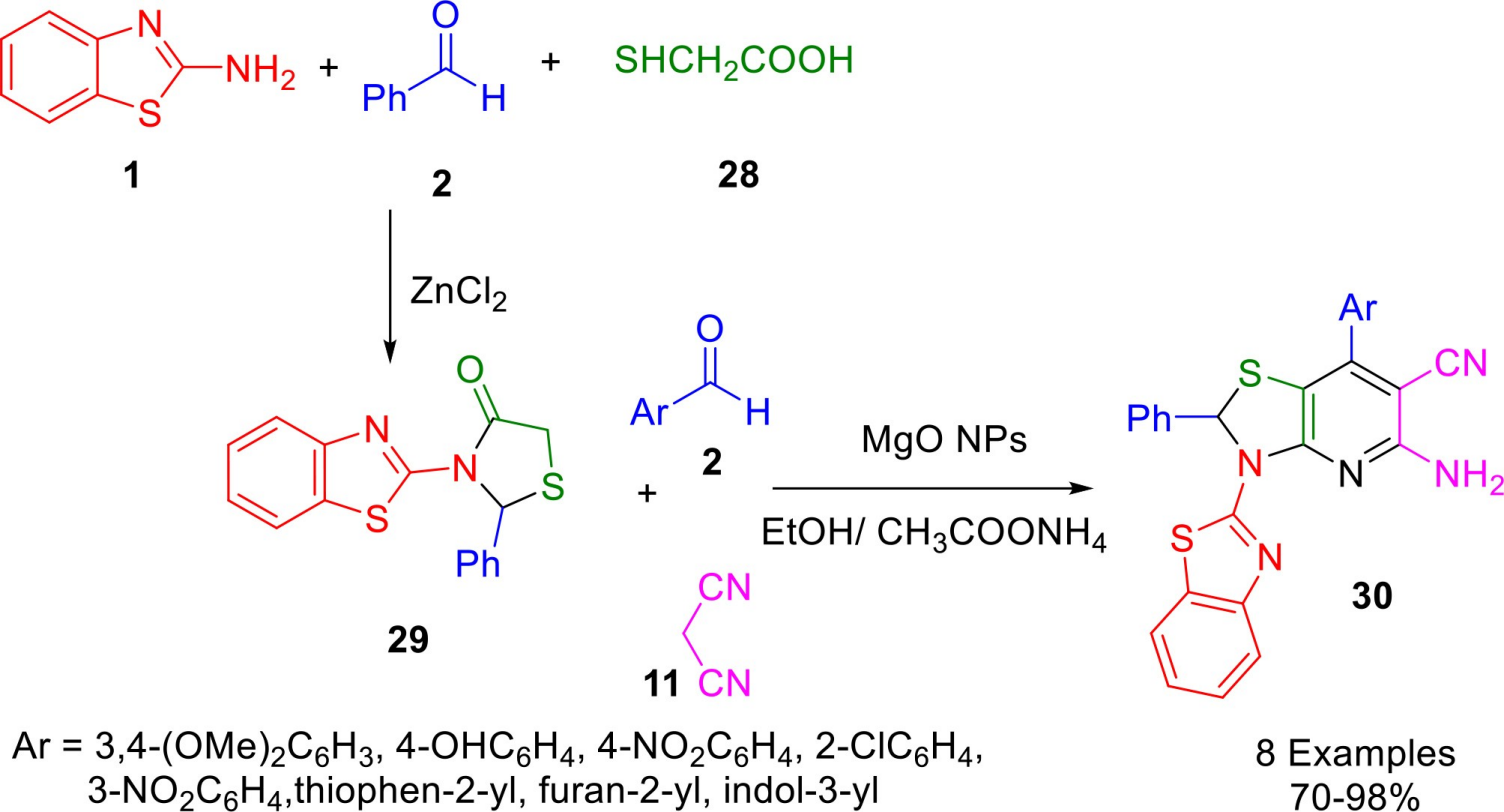
Synthesis of 3‐benzothiazol‐2‐yl‐2‐phenyl‐thiazolidin‐4‐one.

Utilizing green, low‐cost, mild, and efficient magnesium oxide as a heterogeneous base catalyst, Agarwal and Gandhi synthesized 5‐amino‐3‐benzothiazol‐2‐yl‐7‐(phenyl)‐2‐phenyl‐2,3‐dihydro‐thiazolo[4,5‐*b*]pyridine‐6‐carbonitrile (**30**) using a Knoevenagel condensation followed by a Michael addition (Scheme 29). This one‐pot multicomponent reaction uses 2‐aminonezothiazole (**1**), aldehyde (**2**) and 2‐mercaptoacetic acid (**28**) to generate 3‐benzothiazol‐2‐yl‐2‐phenylthiazolidin‐4‐one (**29**) as an intermediate, which then reacts with malononitrile (**11**) and another molecule of aldehyde (**2**) in an environmentally friendly process. Ammonium acetate in ethanol alongside MgO NPs as catalysts were used to create the thiazolo[4,5‐*b*]pyridine‐6‐carbonitrile derivatives. The methodology possesses a number of attractive features, including high product yields, mild reaction conditions, the use of non‐toxic organic solvents, and the ability to recover and reuse the nanocatalyst.^[157]^

**Scheme 30:**
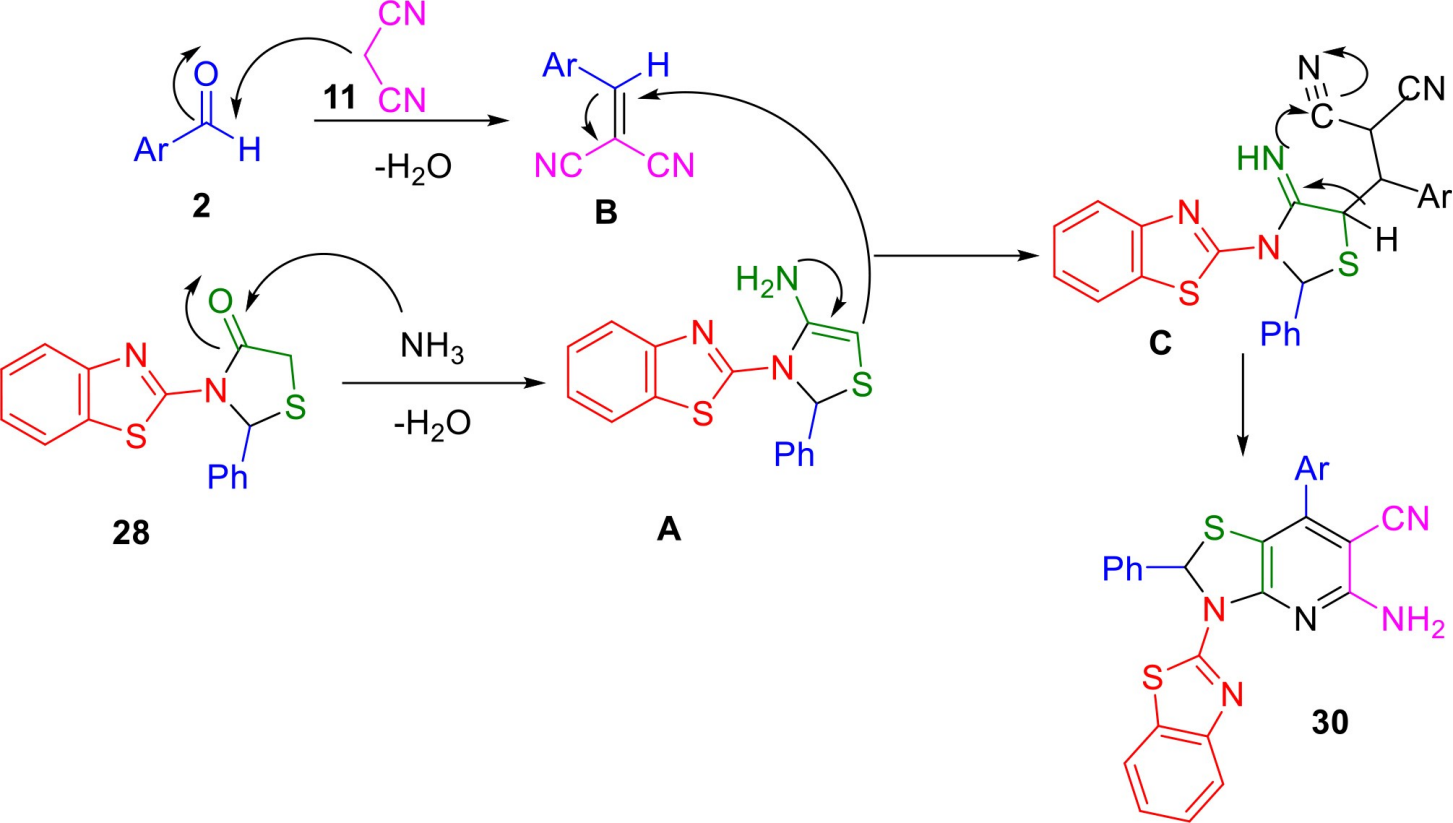
Plausible mechanistic pathway of reaction.

MgO nanoparticles are assumed to activate the carbonyl carbon of 3‐benzothiazol‐2‐yl‐2‐phenyl‐thiazolidin‐4‐one to react with ammonium acetate, resulting in the formation of enamine intermediate **A**. At the same time, a Knoevenagel condensation of substituted aromatic aldehyde (**2**) and malononitrile (**11**) produce arylidene intermediate **B**., The intermediates **A** and **B** then combine, with the activated double bond of the arylidene intermediate **B** being attacked by the nucleophilic endocyclic carbon of enamine intermediate (**A**) in a Michael addition reaction. Following intramolecular cyclization, the adduct is oxidized and aromatized to provide the target compounds (**30**) (Scheme 30).

**Scheme 31:**
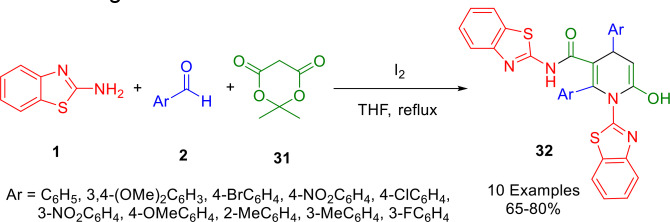
Synthesis of N,1‐bis(benzo[*d*]thiazol‐2‐yl)‐6‐hydroxy‐2,4‐diaryl‐1,4‐dihydropyridine‐3‐carboxamides.

The surfactant‐catalyzed one‐pot approach of Mehrabi and co‐workers for the synthesis of 1,4‐dihydropyridine hybrids (**32**) was reported in 2021. Using THF as the solvent under reflux conditions, Mehrabi and co‐workers combined 2‐aminobenzothiazole (**1**), aryl aldehydes (**2**), and Meldrum's acid (**31**) in the presence of iodine (Scheme 31), in a three‐component reaction. Iodine is a cheap and easily available substance that serves as both a Lewis acid and an oxidant in this case. The reaction is atom‐efficient, provides optimal convergence, and forms a large number of new bonds.^[158]^

## 5 Benzoimidazo Frameworks

**Scheme 32:**
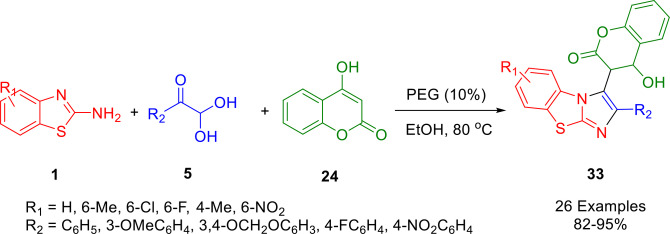
Synthesis of (benzo[*d*]imidazo[2,1‐*b*]thiazol‐3‐yl)‐2*H*‐chromen‐2‐one derivatives.

A simple and environmentally benign one‐pot multicomponent protocol was developed by Jeong *et al*. to efficiently synthesize novel (benzo[*d*]imidazo[2,1‐*b*]thiazol‐3‐yl)‐2*H*‐chromen‐2‐one derivatives (**33**) in excellent yields (Scheme 32). This was achieved using 2‐aminobenzothiazoles (**1**), arylglyoxal monohydrates (**5**) and 4‐hydroxycoumarin (**24**) in ethanol‐PEG‐600. Compared with other approaches that utilize traditional purification techniques, such as chromatography and recrystallization, this protocol has the advantage of utilizing group‐assisited purification (GAP). The reaction also benefits from short reaction times and high conversions. The product represents an important biological scaffold. In designing and synthesizing new drug classes, synthetic and medicinal chemists may use the privileged structural motifs as rich starting points.^[159]^

**Scheme 33:**
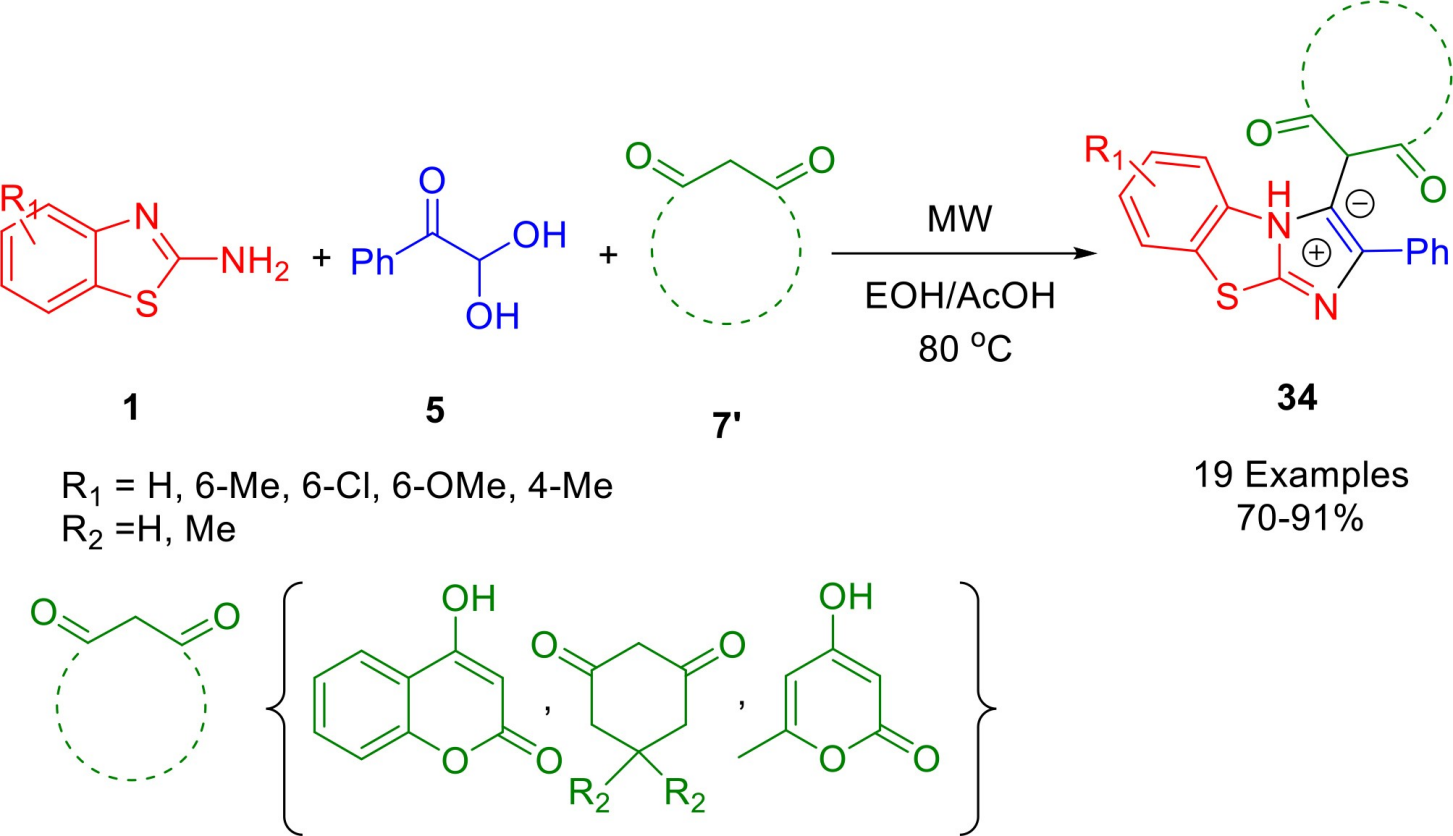
Synthesis of benzo[*d*]imidazo[2,1‐*b*]thiazoles.

Wang and co‐workers synthesized benzo[*d*]imidazo[2,1‐*b*]thiazole derivatives (**34**) *via* an operationally simple and highly efficient one‐pot, multi‐component domino cyclization of 2‐amine‐benzothiazoles (**1**), arylglyoxal (**5**) and cyclic 1,3‐dicarbonyl compounds (**7′**) under microwave irradiation in EtOH/AcOH at 80 °C (Scheme 33). Formation of the benzo[*d*]imidazo[2,1‐*b*]thiazole skeleton and its functionalization proceed smoothly while avoiding the use of a metal catalyst. Furthermore, the procedure provides few by‐products, and a simple purification operation.^[160]^

**Scheme 34:**
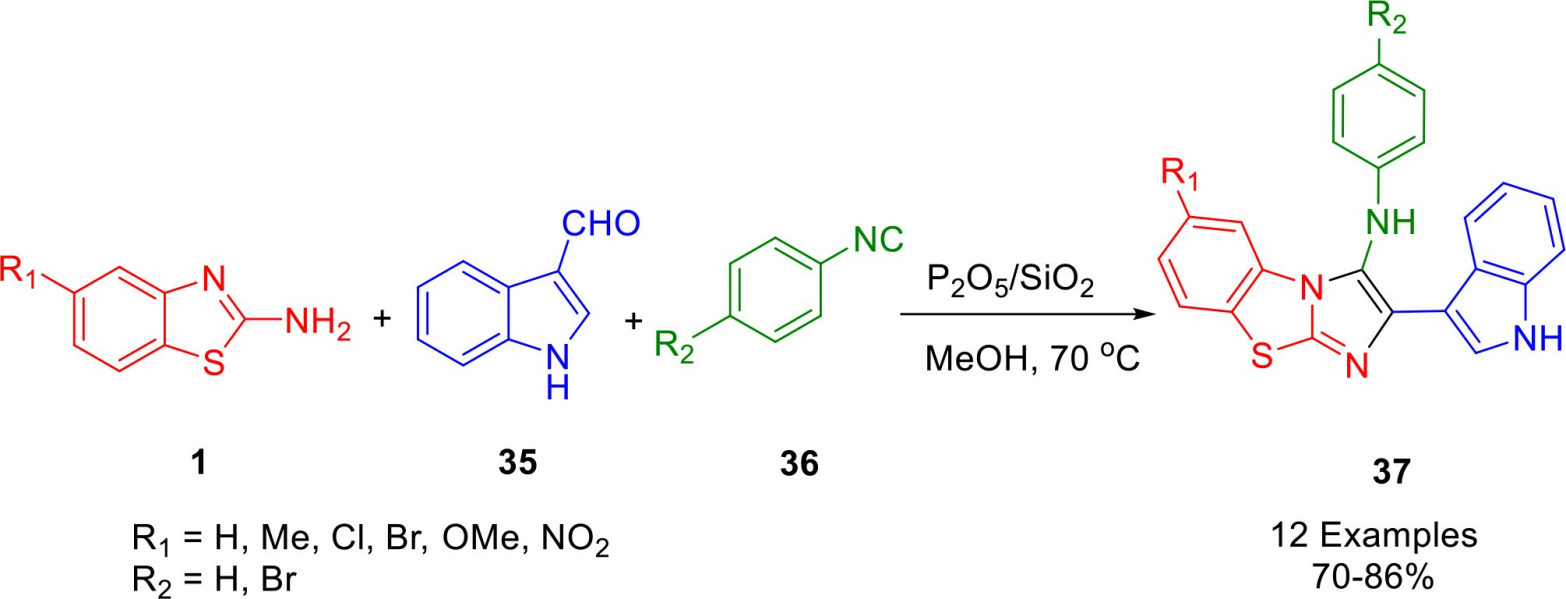
Synthesis of 2‐(1*H*‐indol‐3‐yl)‐*N*‐(phenyl)imidazo[2,1‐*b*][1,3]benzothiazol‐3‐amine derivatives.

A new approach involving a solid supported catalyst for the formation of a C−N bond followed by cyclization has been reported. Yadav and Khan reported a facile, efficient, and environment‐friendly protocol for the synthesis of some new biologically important 3‐amino‐imidazo[2,1‐*b*](1,3)benzothiazole derivatives (**37**) *via* a one‐pot condensation of 2‐aminobenzothiazole (**1**), indole‐3‐carbaldehyde (**35**), and aryl isocyanide (**36**). The reaction was performed in methanol at 70 °C in the presence of silica‐supported P_2_O_5_, which served as a heterogeneous solid acid catalyst. A conventional method was carried out under green conditions (Scheme 34). The present approach offers the advantages of simple methodology, inexpensive acid catalyst, short reaction time, easy work‐up, excellent yields, simple purification and the use of a green solvent. The catalyst was reused four times without any significant loss of its activities in the reactions.^[161]^

**Scheme 35:**
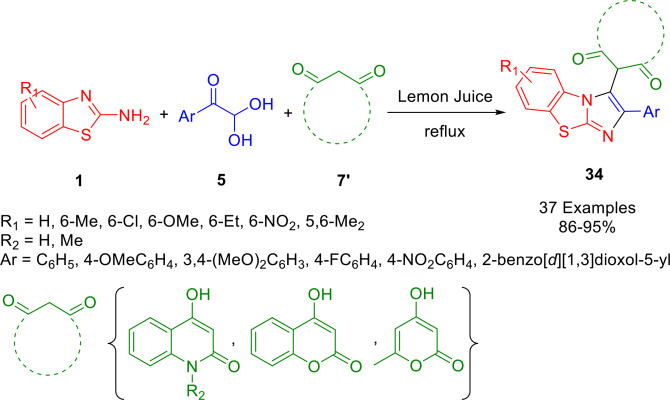
Synthesis of tricyclic fused imidazoles.

As reported by Saha *et al*., various pharmaceutically important tricyclic fused imidazoles (**34**) tethered to aryl and various cyclic 1,3‐dicarbonyl groups were prepared. By reacting 2‐aminobenzothiazoles (**1**) and substituted arylglyoxals (**5**) with cyclic 1,3‐diacarbonyls (**7′**) (such as *N*‐methyl‐4‐hydroxyquinolone, 2,4‐dihydroxyquinoline, 4‐hydroxycoumarin (**24**), or 4‐hydroxy‐6‐methyl‐2‐pyrone) under metal‐free conditions at 110 °C, a variety of tricyclic fused imidazoles with aryl and cyclic 1,3‐dicarbonyl substituents were produced (Scheme 35). Interestingly, lemon juice could be used as the catalyst. Upon checking its reusability, they found that the lemon juice can be recycled three times without affecting its catalytic activity. Performed in an aqueous medium, this method involves a multicomponent reaction catalyzed by natural acids. The various tricyclic fused imidazoles were formed in good to excellent yields.^[162]^

**Scheme 36:**
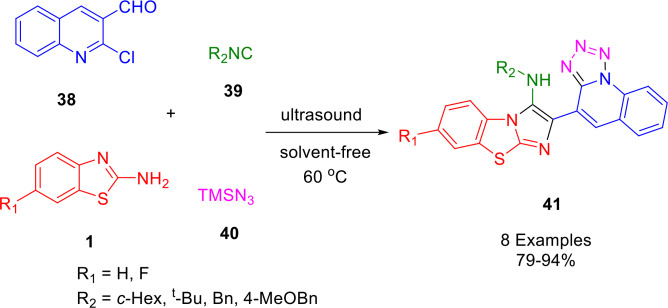
Synthesis of benzo[*d*]imidazo[2,1‐*b*]thiazoles.

Gámez‐Montaño and her research group demonstrated a new one‐pot Groebke−Blackburn−Bienayme reaction (GBBR)/S_N_Ar/ring‐chain azido‐tautomerization strategy for the synthesis of bioactive fused bis‐heterocycles benzo[*d*]imidazo[2,1‐*b*]thiazoles and 1,5‐disubstituted tetrazole (1,5‐DsT) (**41**) (Scheme 36). The desired compounds, formed in an efficient, endogenous water‐triggered MCR, contain a quinoline moiety. Two types of fused heterocycles can be synthesized simultaneously under mild and green conditions. The ultrasound‐assisted four‐component reaction of 2‐chloro‐3‐formylquinoline (**38**), 6‐substituted‐2‐aminobenzothiazole (**1**), and isocyanide (**39**) was followed by adding trimethylsilylazide (**40**) under solvent‐free and catalyst‐free conditions. Yields of 79–94% of the desired products were obtained using ultrasound irradiation (USI) (60 °C, 42 kHz).^[163]^

**Scheme 37:**
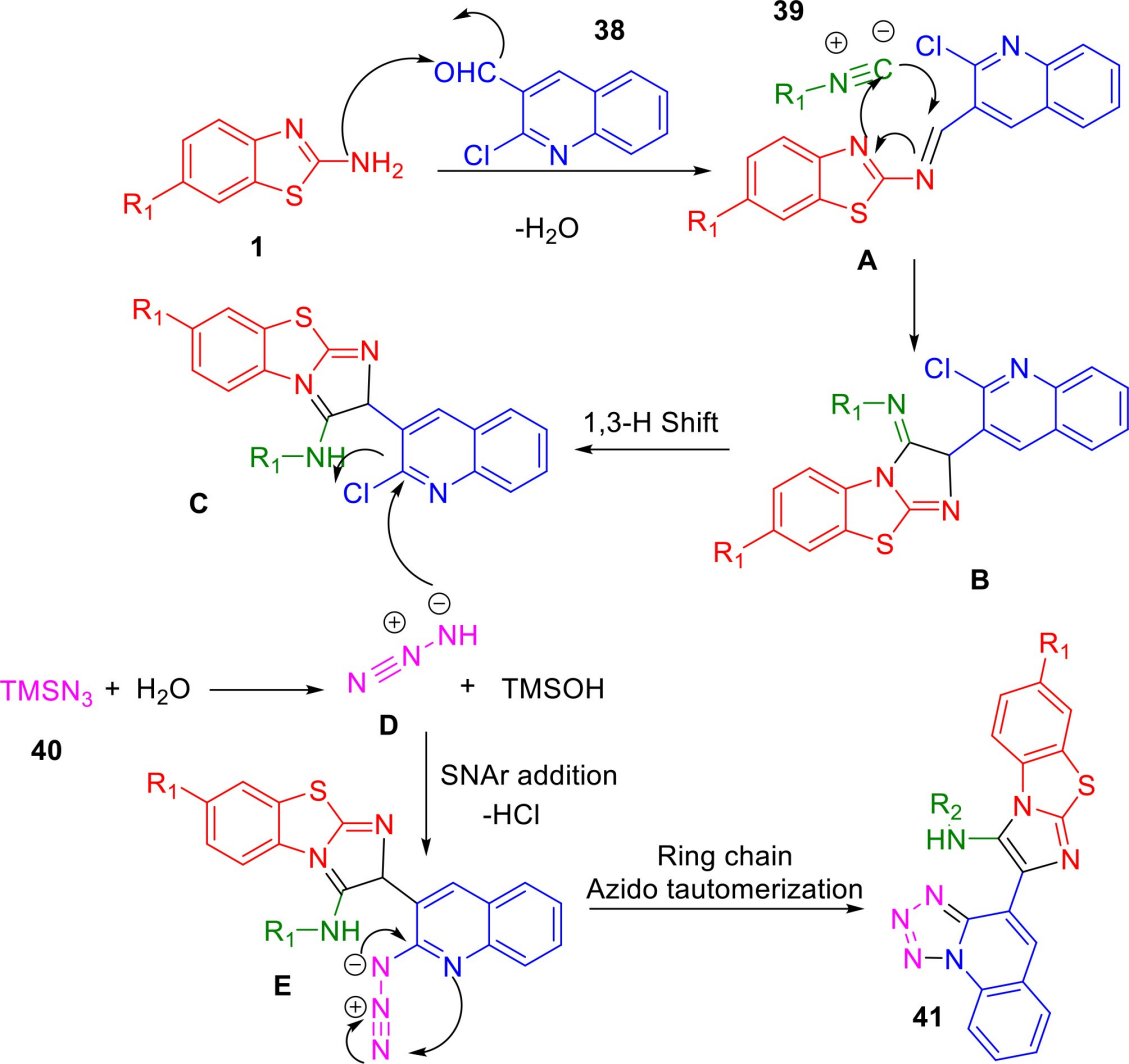
Plausible one‐pot GBBR/S_N_Ar/ring‐chain azido‐tautomerization mechanism.

A plausible mechanism is outlined in Scheme 37. The condensation of 2‐aminobenzothiazole (**1**) with aldehyde (**2**) forms imine **A**, which then undergoes [4+1] cycloaddition with isocyanide (**39)** to form intermediate **B**. This is followed by a spontaneous 1,3‐hydride shift to give the GBBR product **C**. After this 1,3‐*H* shift, the reaction of TMSN_3_ with water (formed during imine condensation) produces hydrazoic acid (HN_3_) **D**. Next, HN_3_ reacts with **C** in an S_N_Ar reaction to generate intermediate **E**. Finally, the azide group undergoes ring‐chain azido‐tautomerization to furnish benzo[*d*]imidazo[2,1‐*b*]thiazoles (**41**).

**Scheme 38:**
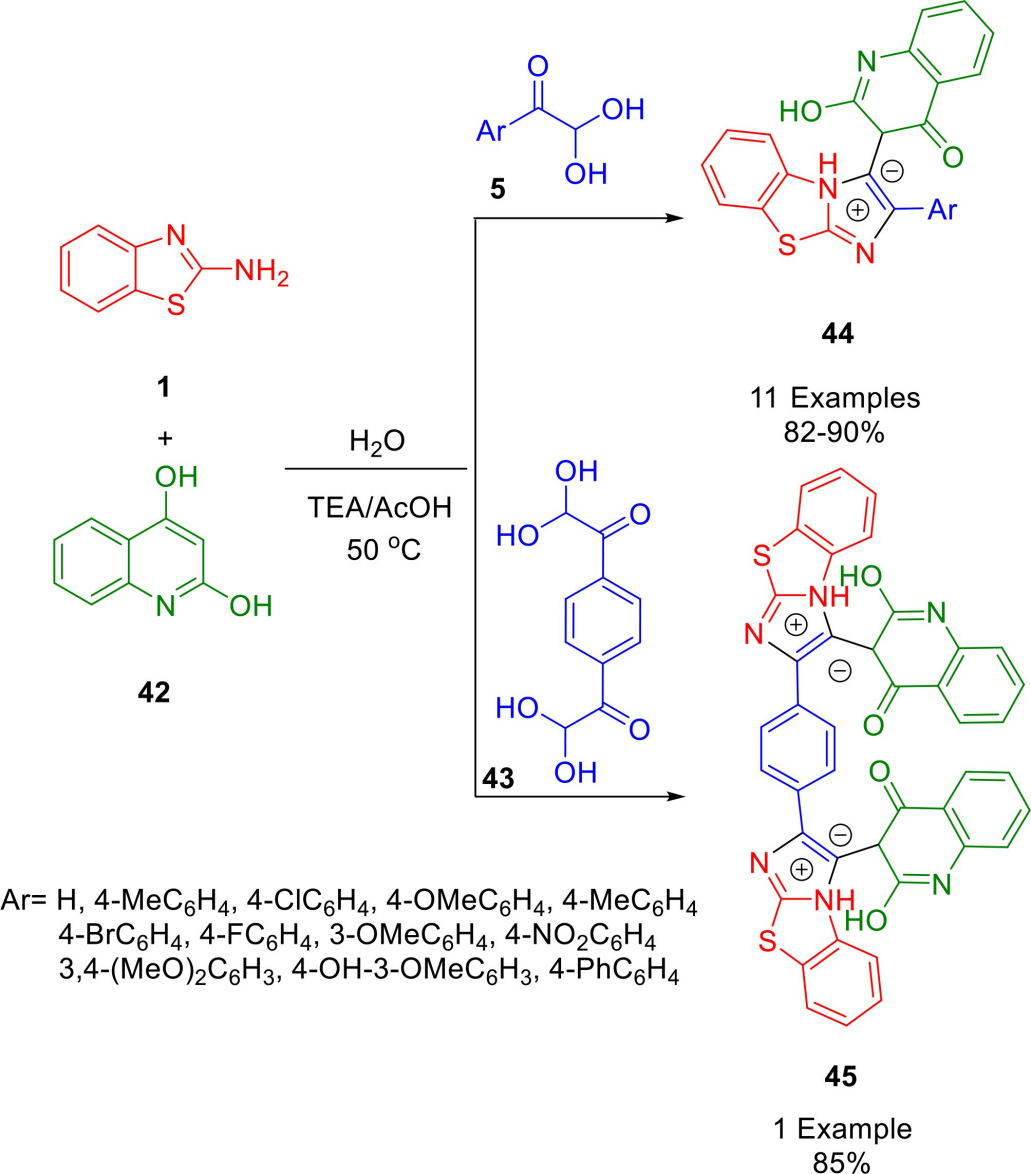
Synthesis of a new series of benzo[*d*]imidazo[2,1‐*b*]thiazole‐1‐ium hydroxides.

In 2019, Etivand *et al*. synthesized novel benzo[*d*]imidazo[2,1‐*b*]thiazole‐1‐ium hydroxide derivatives (**44**, **45**) by combining arylglyoxals (**5**, **43**), quinoline‐2,4‐diols (**42**), and 2‐aminobenzothiazoles (**1**) at 50 °C, using TEA/AcOH (1 : 2) as their catalyst system (Scheme 38). Using this procedure, robust tricyclic benzo[*d*]imidazo[2,1‐*b*]thiazole derivatives with biological and pharmaceutical potential can be produced in high yields under mild reaction conditions. The reaction uses green solvents, and features a simple setup and isolation procedure.^[164]^

**Scheme 39:**
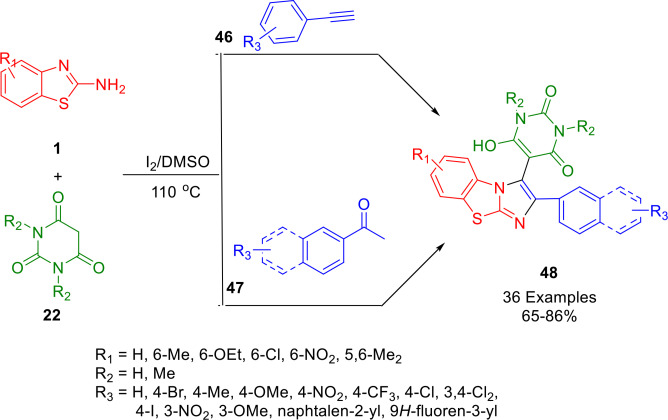
Synthesis of 2‐arylbenzo[*d*]imidazo[2,1‐*b*]thiazoles.

In 2019, Choudhury and coworkers reported another significant achievement – the synthesis of 2‐arylbenzo[*d*]imidazo[2,1‐*b*]thiazoles (**48**) tethered to barbituric acid (**22**). In DMSO medium, 2‐aminobenzothiazoles (**1**), barbituric acids (**22**), and aryl acetylenes (**46**) or aryl methyl ketones (**47**) were reacted in a three‐component reaction (Scheme 39). The MCR can be conducted both conventionally and under microwave irradiation. In this method, one C−C bond and two C−N bonds are formed in one pot by metal‐free oxidation followed by cyclization, leading to the selective formation of an imidazole ring. A large substrate range is offered and the products contain more than one pharmaceutically important motif. The products can be synthesized on gram scale and can be easily purified.^[165]^

**Scheme 40:**
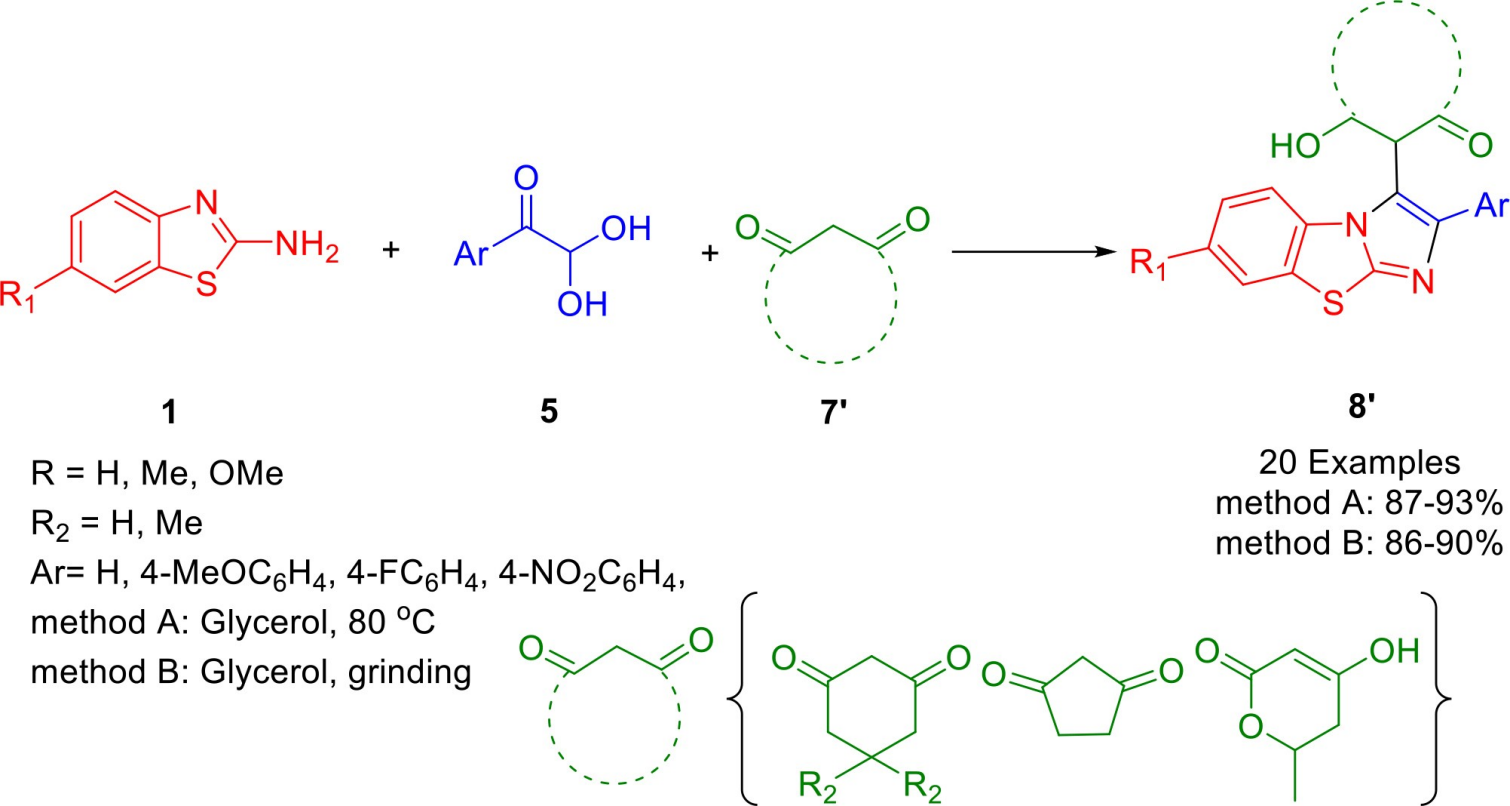
Synthesis of benzo[*d*]imidazo[2,1‐*b*]thiazole derivatives.

In 2019, Khurana and his team demonstrated that one pot, catalyst‐free synthesis of benzo[*d*]imidazo[2,1‐*b*]thiazole derivatives (**8′**) can be acheived using phenylglyoxal (**5**), cyclic enolizable carbonyl compounds (**7′**), and 2‐aminobenzothiazoles (**1**) by combining them at 80 °C or by grinding them at room temperature in glycerol (Scheme 40). As part of this protocol, the following carbonyl compounds are employed: Dimedone, cyclohexa‐1,3‐dione, cyclopenta‐1,3‐dione, 5‐methylcyclohexa‐1,3‐dione, and 4‐hydroxy‐6‐methyl‐2‐pyrone. X‐ray crystallography confirmed the structure of compounds (R_1_=H, Ar=C_6_H_5_, and cyclopentane‐1,3‐dione), and (R_1_=OMe, Ar=C_6_H_5_, and dimedone). There are many advantages to this protocol, including short reaction times, facile work‐ups, high yields, and low environmental impact.^[166]^

**Scheme 41:**
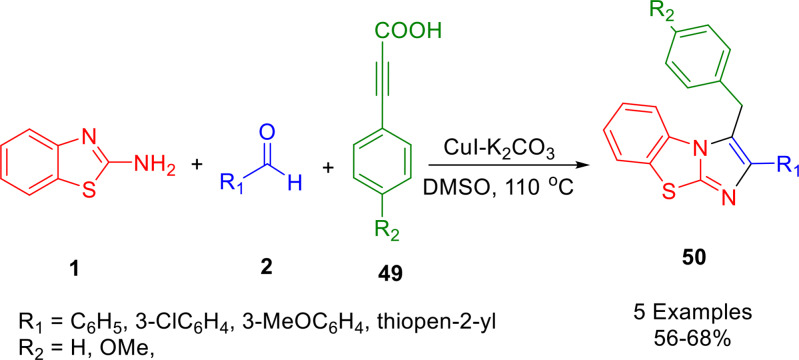
Synthesis of imidazo[2,1‐*b*]benzothiazoles.

The construction of imidazo[2,1‐*b*]benzothiazole derivatives (**50**) was achieved through a straightforward one‐pot MCR involving 2‐aminobenzothiazole (**1**), substituted benzaldehydes (**2**), and 3‐aryl propiolic acids (**49**) (Scheme 41). This reaction, reported by Wu *et al*., uses a catalytic amount of CuI and K_2_CO_3_ to generate the desired compounds in moderate to good yields. The main advantages of this new approach are that it uses readily available starting materials, alkyne sources that are easy to store and handle, the catalysts are common and inexpensive, and there is no requirement for the isolation of intermediates during the reaction. The imidazothiazole derivatives produced in this reaction should have tremendous benefits in medicine, materials, and other important areas.^[167]^

**Scheme 42:**
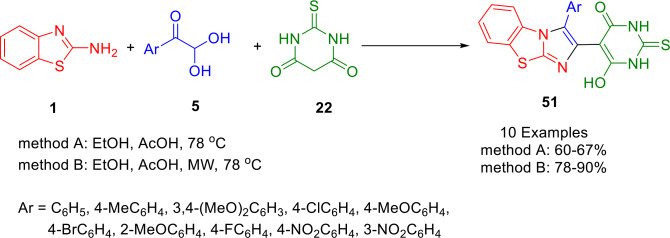
Synthesis of benzo[*d*]imidazo[2,1‐*b*]thiazol‐2‐yl)‐2,3‐dihydropyrimidin‐4(1*H*)‐ones.

Asadi *et al*. described a convenient and effective protocol for the synthesis of benzimidazo[2,1‐*b*]thiazole derivatives (**51**) *via* the one‐pot, three‐component reaction of different arylglyoxals (**5**), thiobarbituric acid (**22**), and 2‐aminobenzothiazole (**1**) in the presence of acetic acid in ethanol under both thermal and microwave conditions (Scheme 42).^[168]^

## 6 Aminobenzothiazole Frameworks

**Scheme 43:**
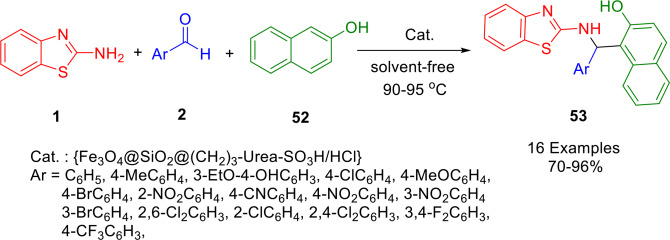
Synthesis of 2′‐aminobenzothiazolomethylnaphthol derivatives.

Zolfigol *et al*. demonstrated the catalytic performance of a magnetically recoverable solid acid in a one‐pot cascade reaction that produced 2′‐aminobenzothiazolomethylnaphthol derivatives (**52**) using a mixture of aromatic aldehydes (**2**), 2‐naphthol (**51**) and 2‐aminobenzothiazole (**1**), in the presecne of Fe_3_O_4_@SiO_2_@(CH_2_)_3_‐Urea‐SO_3_H/HCl as a nanomagnetic catalyst under solvent‐free conditions (Scheme 43). The applied nanomagnetic core–shell catalyst demonstrated robust activity in the preparation of the target molecules. This synthetic protocol demonstrated that Fe_3_O_4_@SiO_2_@(CH_2_)_3_‐Urea‐SO_3_H/HCl was extremely recoverable through separation with an external magnet. Furthermore, the recovered catalyst could be reused up to eight times. The simplicity of the reaction procedures, high to excellent yields, purity of the target molecules, robust activity of the catalysts, and a clean reaction profile are the prominent features of the reported protocol.^[169]^

**Scheme 44:**
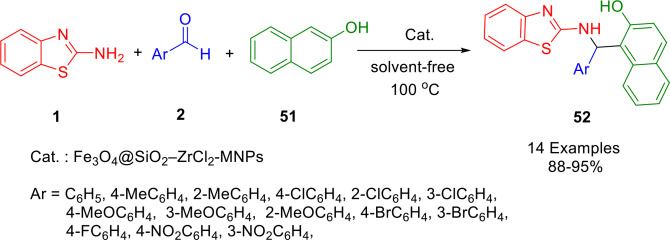
Synthesis of 1‐(benzothiazolylamino)methyl‐2‐naphthol derivatives.

An alternative green, simple and eco‐friendly tandem condensation process for the synthesis of 1‐(benzothiazolylamino)methyl‐2‐naphthol (**52**) has been described by Shirini and Kamali, who used novel magnetic nanoparticles formulated as Fe_3_O_4_@SiO_2_‐ZrCl_2_‐MNPs, in the condensation of aryl aldehydes (**2**), *β*‐naphthol (**51**) and 2‐aminobenzothiazole (**1**) under solvent‐free conditions (Scheme 44). The method provides certain economic and environmental advantages, including a clean procedure, solvent‐free conditions, a relatively short reaction time, and high yields. Furthermore, an external magnet can be used to recover the catalyst several times while maintaining consistent activity.^[170]^

**Scheme 45:**
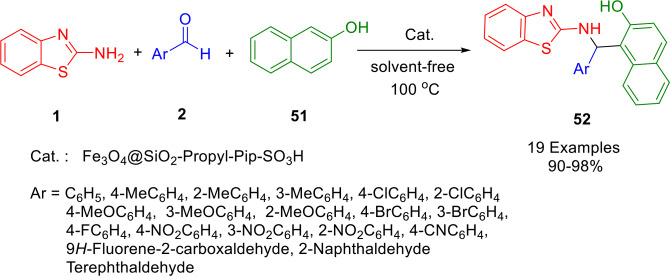
Synthesis of 1‐(benzothiazolylamino)phenylmethyl‐2‐naphthol derivatives.

In another study, Shirini and co‐workers demonstrated an efficient photochemical synthesis of 1‐(benzothiazolylamino) phenylmethyl‐2‐naphthols (**52**) *via* a one‐pot condensation of aldehydes (**2**), 2‐aminobenzothiazole (**1**), and 2‐naphthol (**51**) in the absence of solvent (Scheme 45). 4‐(4‐Propylpiperazine‐1‐yl)butane‐1‐sulfonic acid‐modified silica‐coated magnetic nanoparticles have been synthesized *via* covalent grafting of piperazine on (3‐chloropropyl)triethoxysilane‐functionalized magnetic nanoparticles through a ring‐opening reaction on 1,4‐butane sultone. Mechanical decantation using an external magnet makes it easy to isolate the catalyst from the reaction mixture and then reuse it up to seven times without significantly deteriorating its performance.^[171]^

**Scheme 46:**
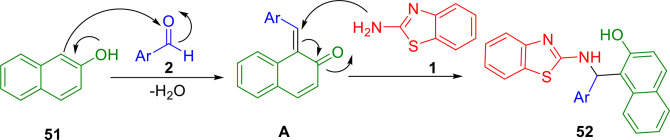
Proposed mechanism for the synthesis of 1‐(benzothiazolylamino) phenylmethyl‐2‐naphthols.

A proposed mechanism for the studied reactions mediated by Fe_3_O_4_@SiO_2_‐Propyl‐Pip‐SO_3_H⋅HSO_4_ is shown in Scheme 46. On the basis of this mechanism, Fe_3_O_4_@SiO_2_‐Propyl‐Pip‐SO_3_H⋅HSO_4_ catalyzes various reaction steps by acting as a Lewis acidic reagent.

**Scheme 47:**
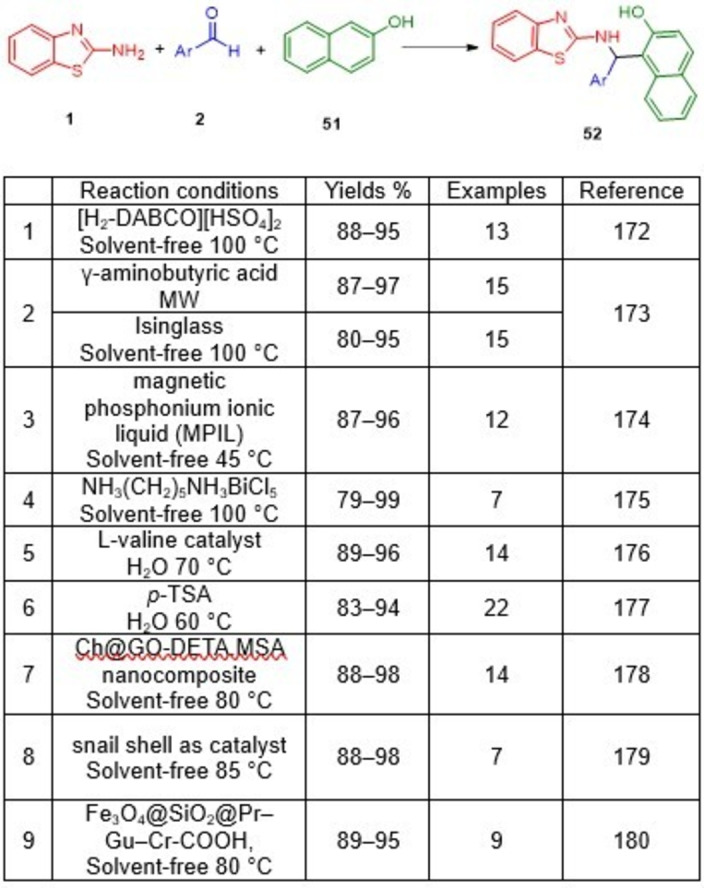
Different methods for the synthesis of 1‐(benzothiazolylamino)phenylmethyl‐2‐naphthol derivatives.

Various types of catalysts have been used, as well as some catalyst‐free processes, to develop a number of methods for accessing compound **52**. The results for combining aldehydes (**2**), 2‐aminobenzothiazole (**1**), and 2‐naphthol (**51**), to form 1‐(benzothiazolylamino)phenylmethyl‐2‐naphthol derivative (**52**) are summarized in Scheme 47.

## 7 Spiro Frameworks

**Scheme 48:**
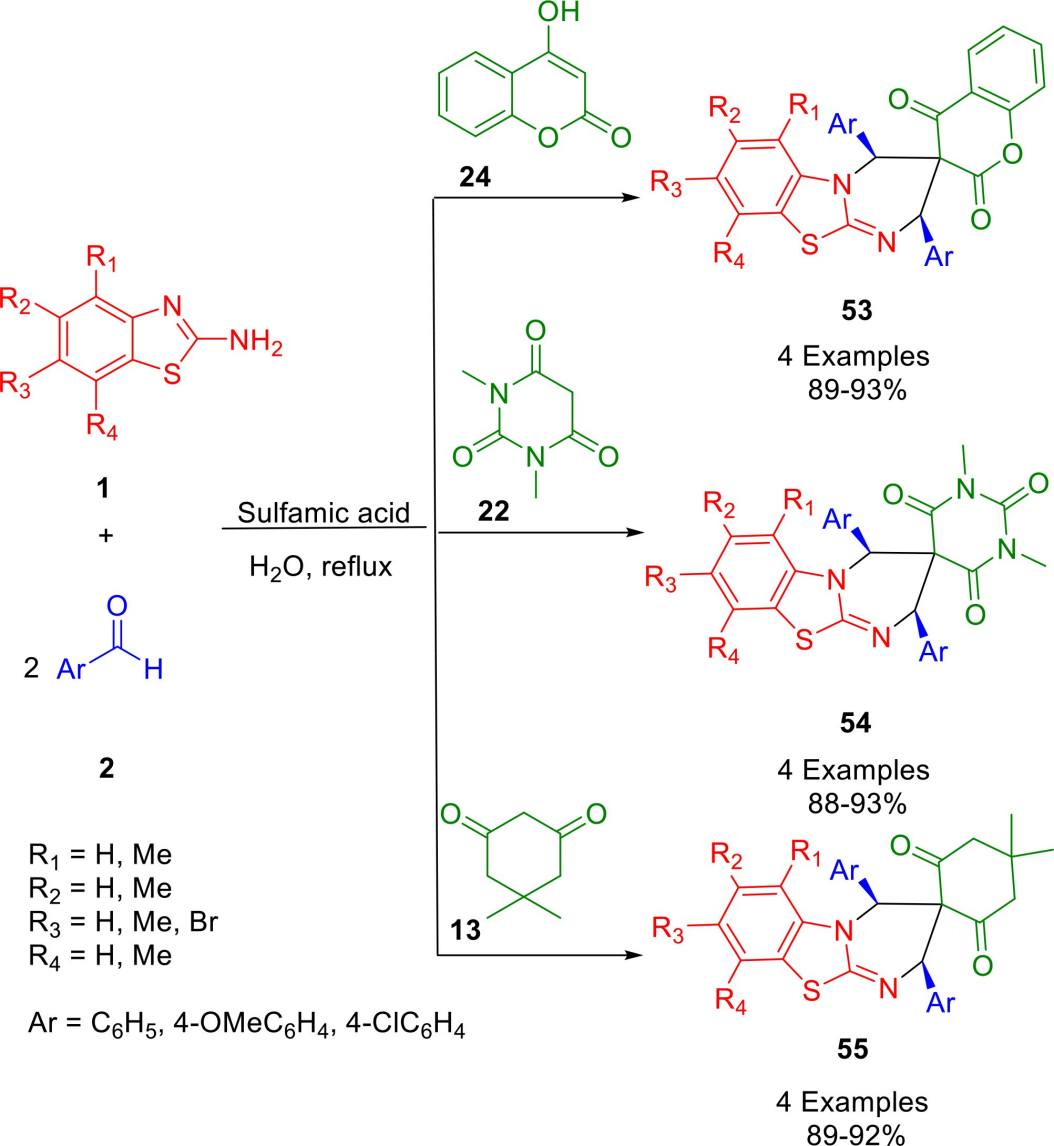
Synthesis of spiroheterocycles.

The research group of Arya reported an environmentally benign, efficient, and facile synthesis of structurally diverse spiroheterocycles (spiro[pyrimido[2,1‐*b*]benzothiazole‐3,3′‐chromene]‐2′,4′‐dione (**53**), spiro[pyrimido[2,1‐*b*]benzothiazole‐3,5′‐pyrimidine]‐2′,4′,6′‐triones (**54**), and spiro[pyrimido[2,1‐*b*]benz‐thiazole‐3,2′‐cyclohexane]‐1′,3′‐diones (**55**)) *via* a one‐pot, pseudo‐four component reaction of 2‐aminobenzothiazoles (**1**) with aromatic aldehydess (**2**) and cyclic *β*‐diketones (**13**, **22**, **24**) in aqueous medium (Scheme 48). Spiroheterocycles can be easily fused with biologically active scaffolds by hetero‐Diels–Alder cycloadditions. Calculations using density functional theory have determined the configuration of the hetero‐Diels‐Alder cycloadducts.^[181]^

**Scheme 49:**
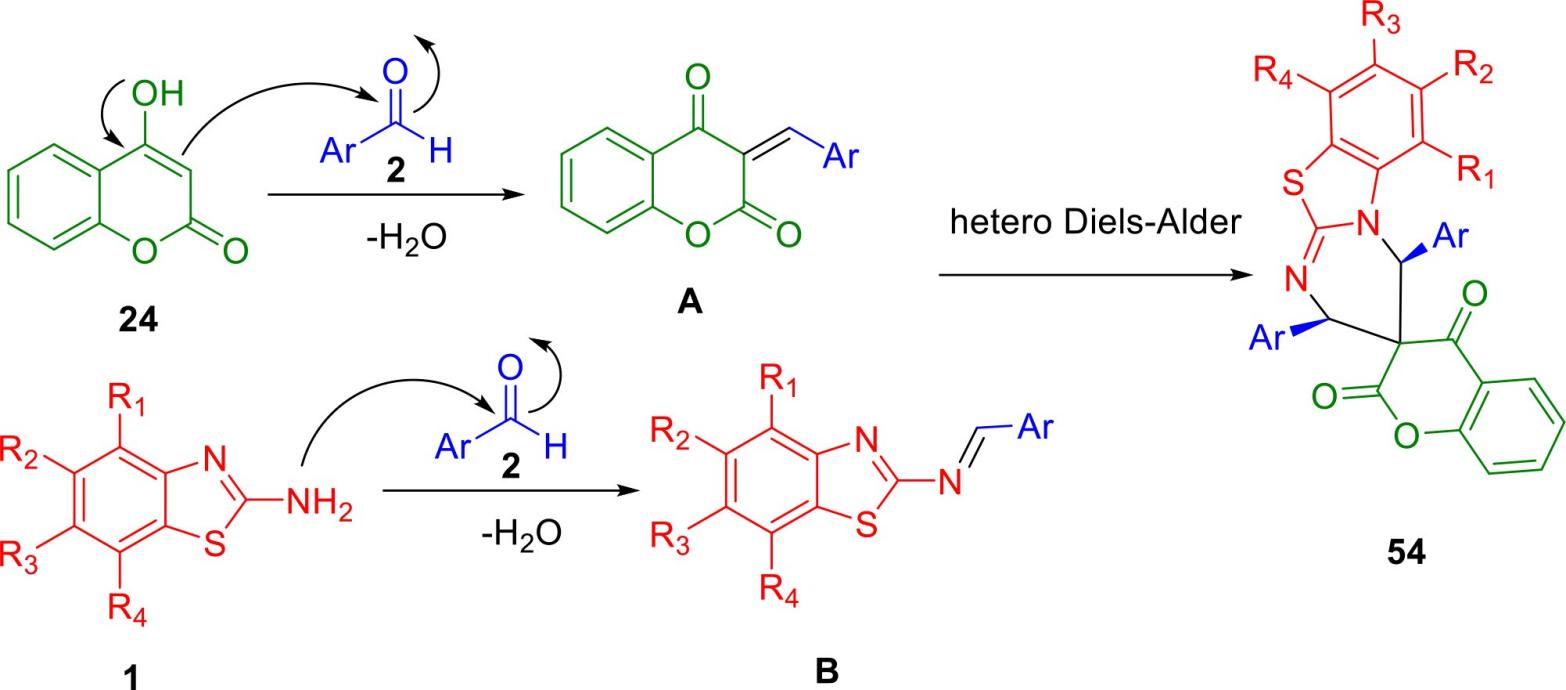
Plausible mechanism for the synthesis of spiroheterocycles.

Scheme 49 illustrates the proposed mechanism for the reaction. This reaction occurs through the acid catalyzed condensation of cyclic diketone with aromatic aldehyde (**2**) to produce the intermediate **A**. 2‐aminobenzothiazole (**1**) is condensed with the second molecule of aromatic aldehyde (**2**) to form the imine **B** in the presence of acid. Spiro[pyrimido]benzothiazole‐3,3′‐chromene]‐2′,4′‐diones (**54**) are then produced from the hetero‐Diels‐Alder reaction between **A** and **B**. The synthesis of spiro[pyrimido[2,1‐*b*]benzothiazole‐3,3′‐chromene]‐2′,4′‐diones therefore requires two equivalents of aldehyde (**2**).

**Scheme 50:**
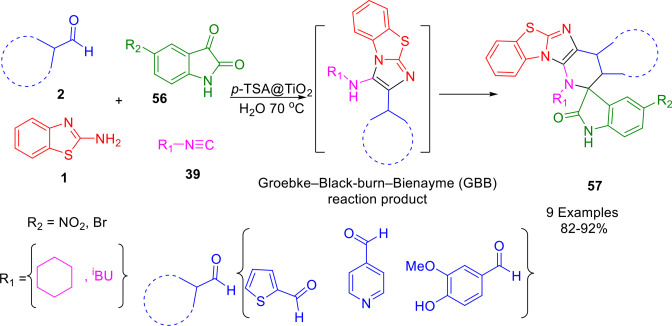
synthesis of spirooxindoles.

A synthetic protocol utilizing multicomponent isocyanide reactions and modified TiO_2_ nanoparticles containing *p*‐TSA as a heterogeneous acid catalyst in aqueous media was developed by Kumar and colleagues to synthesize highly functionalized spirooxindole analogues (**57**) (Scheme 50). To synthesize the spiro compound, the Groebke–Blackburn–Bienayme (GBB) reaction product was first obtained through the reaction of 2‐aminobenzothiazole (**1**), aldehyde (**2**) and isocyanide (**39**), and then, after separating the GBB product, it reacted with isatin (**56**) to produce the final product. A spiro product with a higher yield was prepared using a post‐modification Pictet‐Spengler reaction using 2‐aminobenzothiazole (**1**), aldehyde (**2**), isocyanide, and isatin during a single step without isolation (GBB). After recovery, the catalyst could be reused eight times while retaining its catalytic activity.^[182]^

This reported protocol for the synthesis of spirooxindoles presents some attractive characteristics: (i) A particular feature of surface‐modified TiO_2_ nanoparticles is that they include an organic/inorganic nanocomposite material that has unique catalytic properties; (ii) the inductive effect of the S=O bonds on the TiO_2_ nanoparticle surface results in the development of both Lewis and Bronsted acid sites; (iii) moreover, the present protocol is environmentally benign, since the catalyst is non‐toxic and reusable and the reactions occur in water (a safe, inexpensive, and green solvent); iv) the reaction produces drug‐like molecules that are structurally diverse and molecularly complex.

## 8 Miscellaneous Compounds

**Scheme 51:**
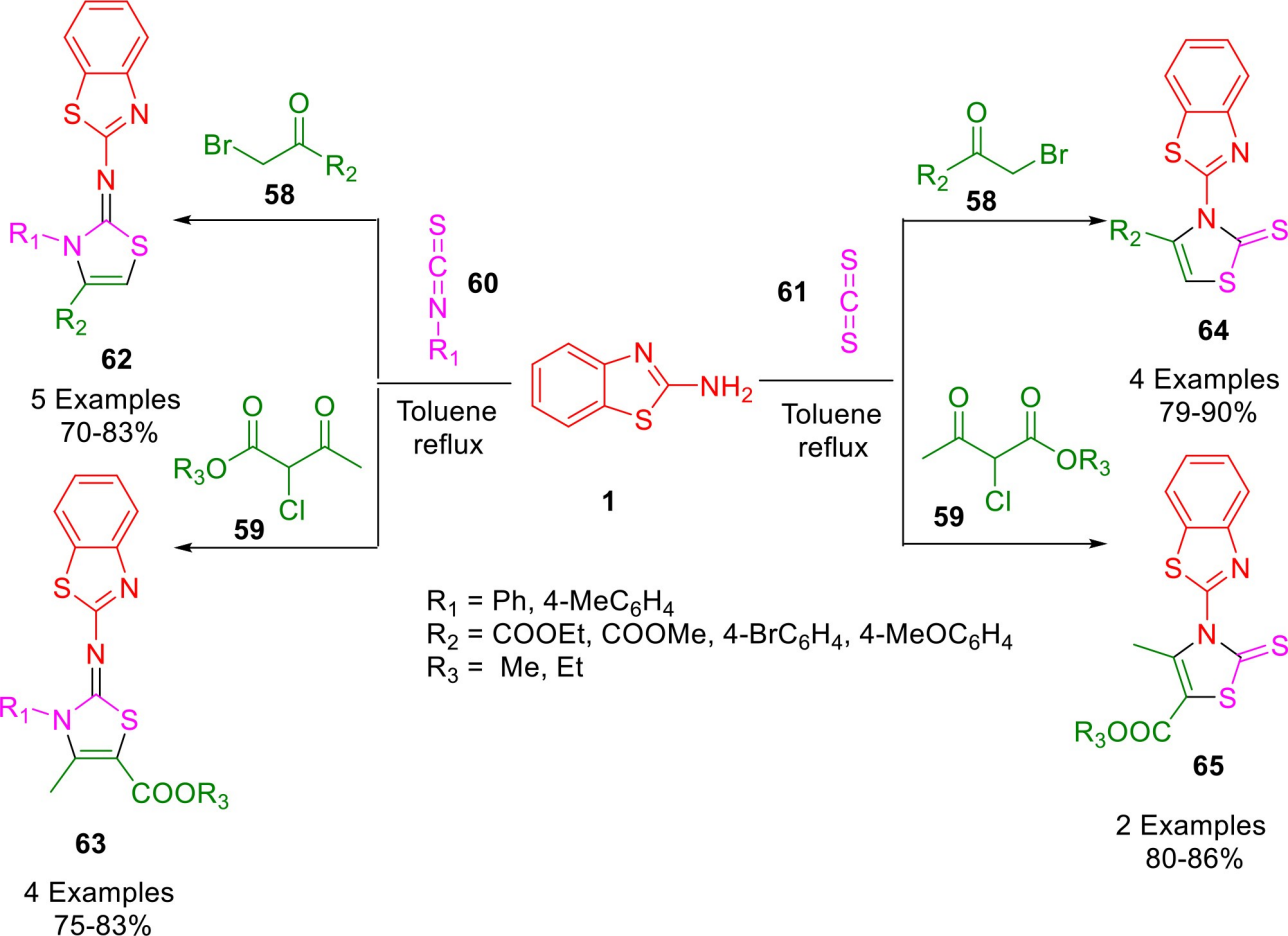
Synthesis of novel bis‐thiazole derivatives.

Arab‐Salmanabadi developed a facile approach for the construction of novel functionalized bis‐thiazole heterocycles (**62**–**65**) in good to excellent yields (70–90 %) *via* the ring closure of 2‐aminobenzothiazole (**1**) and various α‐haloketones (**58**, **59**) in the presence of carbon disulfide (**61**) or aryl isothiocyanates (**60**) as S‐nucleophiles in toluene under refluxing conditions (Scheme 51). The procedure described provides an efficient one‐pot multicomponent methodology for constructing novel functionalized bis‐thiazoles. The high to excellent yields of products without any need for activation, the ready availability of the starting materials, the reaction's simplicity, and its mild conditions are the main advantages of these methods. Considering the wide range of pharmaceutical activities reported for compounds containing a thiazole ring, it is worth investigating the potential pharmaceutical properties of these novel bis‐thiazole derivatives further.^[183]^

**Scheme 52:**
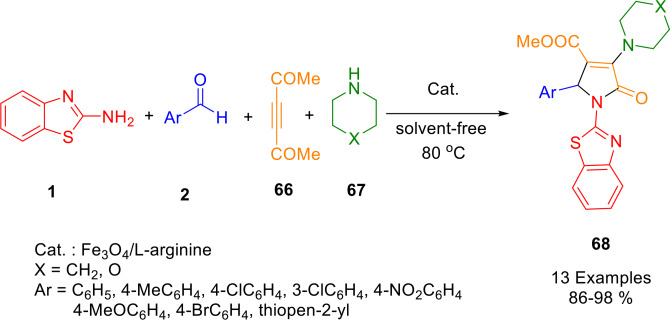
Synthesis of 2‐pyrrolidinone derivatives.

In a three‐component, domino‐type reaction reported by Ghasemzadeh *et al*., functionalized polysubstituted 2‐pyrrolidinone derivatives (**68**) were synthesized regioselectively using Fe_3_O_4_@L‐arginine NPs as reusable organocatalysts at 80 °C (Scheme 52). A simple and green method for synthesizing functionalized biologically active heterocycles (**68**) was developed using a mixture of 2‐aminobenzothiazole (**1**), dimethylacetylenedicarboxylate (**66**), aromatic aldehydes (**2**), and piperidine/morpholine (**67**). By using various spectroscopic techniques, the magnetic nanocatalyst structure of Fe_3_O_4_@L‐arginine nanoparticles was revealed. The advantages of this method are short reaction times, excellent yields, simple work‐up procedures, and reusability of the nanocatalyst (Fe_3_O_4_@L‐arginine nanoparticles), which could be reused six times consecutively, according to the reusability study.^[184]^

**Scheme 53:**
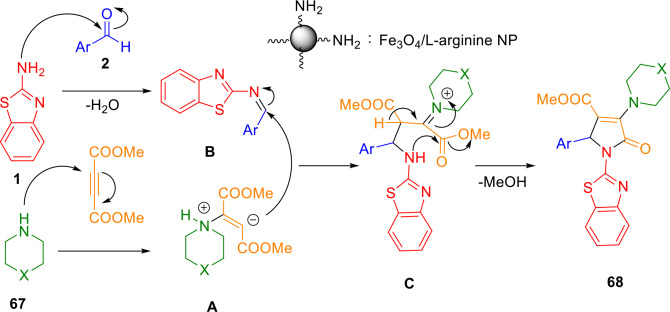
The proposed mechanism for the synthesis of polyfunctionalized 2‐pyrrolidinones.

Based on previously reported procedures^[20]^ and experimental results, Scheme 53 illustrates a plausible mechanism for this MCR. Coating Fe_3_O_4_ with arginine results in NH_2_ groups of the surface, which act as Bronsted base in the dehydration of substrates and intermediates.[^42^, ^43^] Initial reaction of morpholine/piperidine (**67**) with dimethyl acetylenedicarboxylate (**66**) results in intermediate **A** Then, intermediate **B** is formed when 2‐aminobenzothiazole (**1**) nucleophilically attacks the aldehyde. In the next step, intermediate **A** combines with intermediate **B**, thereby forming intermediate **C**, which is then intramolecularly cyclized to form the desired molecules (**68**).

**Scheme 54:**
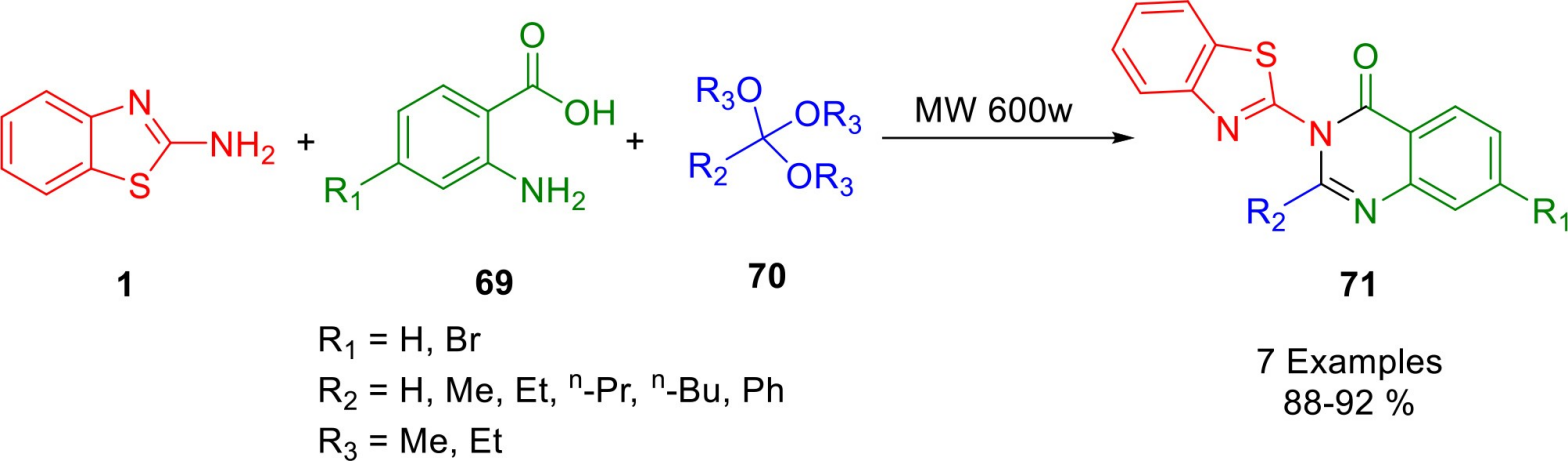
Synthesis of 3‐(benzo[*d*]thiazol‐2‐yl)‐2‐alkyl‐4(3*H*)‐quinazolinones.

Through the condensation of anthranilic acids (**69**), 2‐aminobenzothiazole (**1**), and orthoesters (**70**) under microwave irradiation, Koroji *et al*. demonstrated the utility of silica sulfuric acid in catalyzing the one‐pot synthesis of novel quinazolin‐4(3*H*)‐ones and 3‐(2‐benzimidazolyl)‐2‐alkyl quinazolin‐4(3*H*)‐ones (**71**) as a heterogeneous catalyst (Scheme 54). In addition to providing high yields of products, the method can also be reused, resulting in an easy experimental procedure.^[185]^

## 9 Conclusions

Over the past few decades, the use of 2‐aminobenzothiazole in the multicomponent synthesis of heterocycles has gained significant attention in organic chemistry. This compound has demonstrated versatility and efficiency in the construction of diverse heterocyclic frameworks. These are key structural motifs in many biologically active compounds and pharmaceuticals. 2‐aminobenzothiazole's unique reactivity has enabled its application in the synthesis of various heterocyclic systems, including benzothiazole‐fused heterocycles, benzothiazole‐containing alkaloids, and other complex heterocyclic scaffolds. These advancements have created new avenues for the rapid and efficient synthesis of diverse heterocyclic compounds. This makes 2‐aminobenzothiazole an indispensable tool for organic chemists. This paper has provided an overview of the recent advances in the application of 2‐aminobenzothiazole to the multicomponent synthesis of heterocycles, highlighting their significance and potential in organic synthesis.

## Conflict of Interests

The authors declare no conflict of interest.

10

## Biographical Information

*Ramin Javahershenas was born in Urmia, Iran, in 1971. He received his BSc in applied chemistry from Tabriz University, Tabriz, Iran in 1993, his MSc in organic chemistry from Urmia University, Urmia, Iran under the supervision of Professor Naser Ardabilchi in 1999, and his PhD in organic chemistry from Urmia University, Urmia, Iran under the supervision of Professor Jabbar Khalafy in 2017. His research interests center around organic synthesis and include heterocyclic synthesis, asymmetric synthesis, natural products synthesis, synthetic methodology, and applications of various catalysts in multicomponent reactions*.

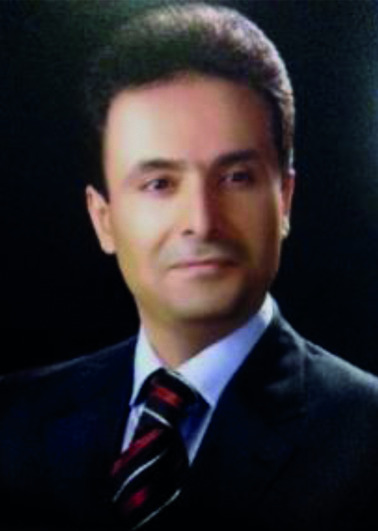

## Biographical Information

*Jianlin Han received his Ph.D. in organic Chemistry in 2007 from Nanjing University. He then carried out postdoctoral studies for one year at Texas Tech University. In 2008, he moved to the University of Oklahoma to continue postdoctoral research for nearly one year. In 2009 he took the position of Associate Professor at the Nanjing University. In 2019, he moved to Nanjing Forestry University and became a professor there. His research topics include organic fluorine chemistry, radical reaction, and asymmetric synthesis*.

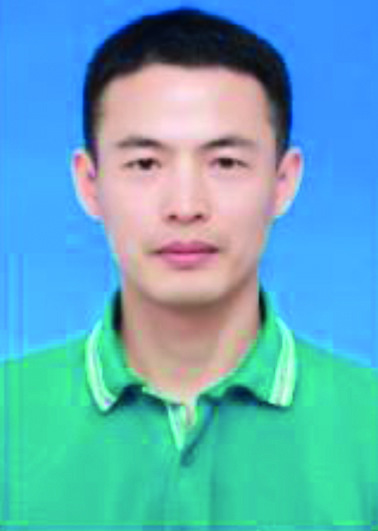

## Biographical Information

*Mosstafa Kazemi was born in Ilam, Iran. He has received MS degree in organic chemistry from Ilam University in 2013, his Ph.D. degree in organic chemistry from Ilam University in 2018. Dr. Kazemi is interested in the development of novel synthetic methods, nanocatalysts and particularly involving the application of Magnetic nanocatalysts in chemical reactions*.

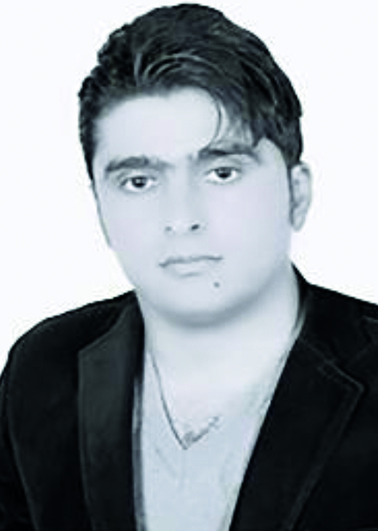

## Biographical Information

*Peter Jervis obtained his master's degree in chemistry in 2004 from the University of Leicester (UK), before completing his PhD in organic chemistry in 2008 at the University of Birmingham (UK), under the supervision of Dr, Liam R. Cox. He then completed postdoctoral work in the laboratory of Prof. Gurdyal S. Besra in glycolipid research, before spending some time working in industry on drug discovery projects for GlaxoSmithKline, Concept Life Sciences and AstraZeneca. He returned to academia in 2018 to participate in a collaboration between the University of Porto and the University of Minho, Portugal. His research interests are organic chemistry, total synthesis, synthetic methodology and chemical biology, alongside the development of supramolecular peptide hydrogels. In these areas, he has published 36 research papers in international peer‐reviewed journals*.

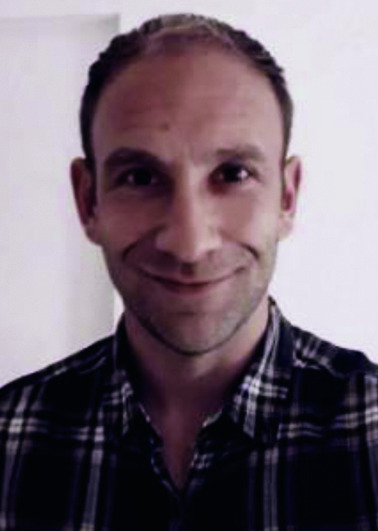

## Data Availability

Research data are not shared.
